# Supplementary material for: Spatial analysis with SPIAT and spaSim to characterize and simulate tissue microenvironments
Source: Nat Commun. 2023 May 15;14:2697. doi: 10.1038/s41467-023-37822-0 (PMC10185501; doi:10.1038/s41467-023-37822-0)
Supplement: Supplementary file 1 — Supplementary Information [file 41467_2023_37822_MOESM1_ESM.pdf]

SUPPLEMENTARY INFORMATION

Spatial analysis with SPIAT and spaSim to characterize and simulate tissue microenvironments

Yuzhou Feng[1], Tianpei Yang[1], John Zhu[1], Mabel Li[1], Maria Doyle[1], Volkan Ozcoban[1], Greg Bass[2], Angela Pizzolla[1,3], Lachlan Cain[1], Sirui Weng[1,3], Anupama Pasam[1], Nikolce Kocovski[1], Yu-Kuan Huang[1,3], Simon P. Keam[1,3], Terence P. Speed[4], Paul J. Neeson[1,3], Richard B. Pearson[1,3], Shahneen Sandhu[1,3], David L. Goode[1,3], Anna S. Trigos[1,3*]

[1]Peter MacCallum Cancer Centre, Melbourne, VIC ,Australia
[2]Research & Development, CSL Innovation, Parkville, VIC, Australia
[3]The Sir Peter MacCallum Department of Oncology, The University of Melbourne, Melbourne, VIC, Australia
[4]Bioinformatics Division, The Walter and Eliza Hall Institute of Medical Research, Parkville, VIC, Australia

*Corresponding author email: anna.trigos@petermac.org

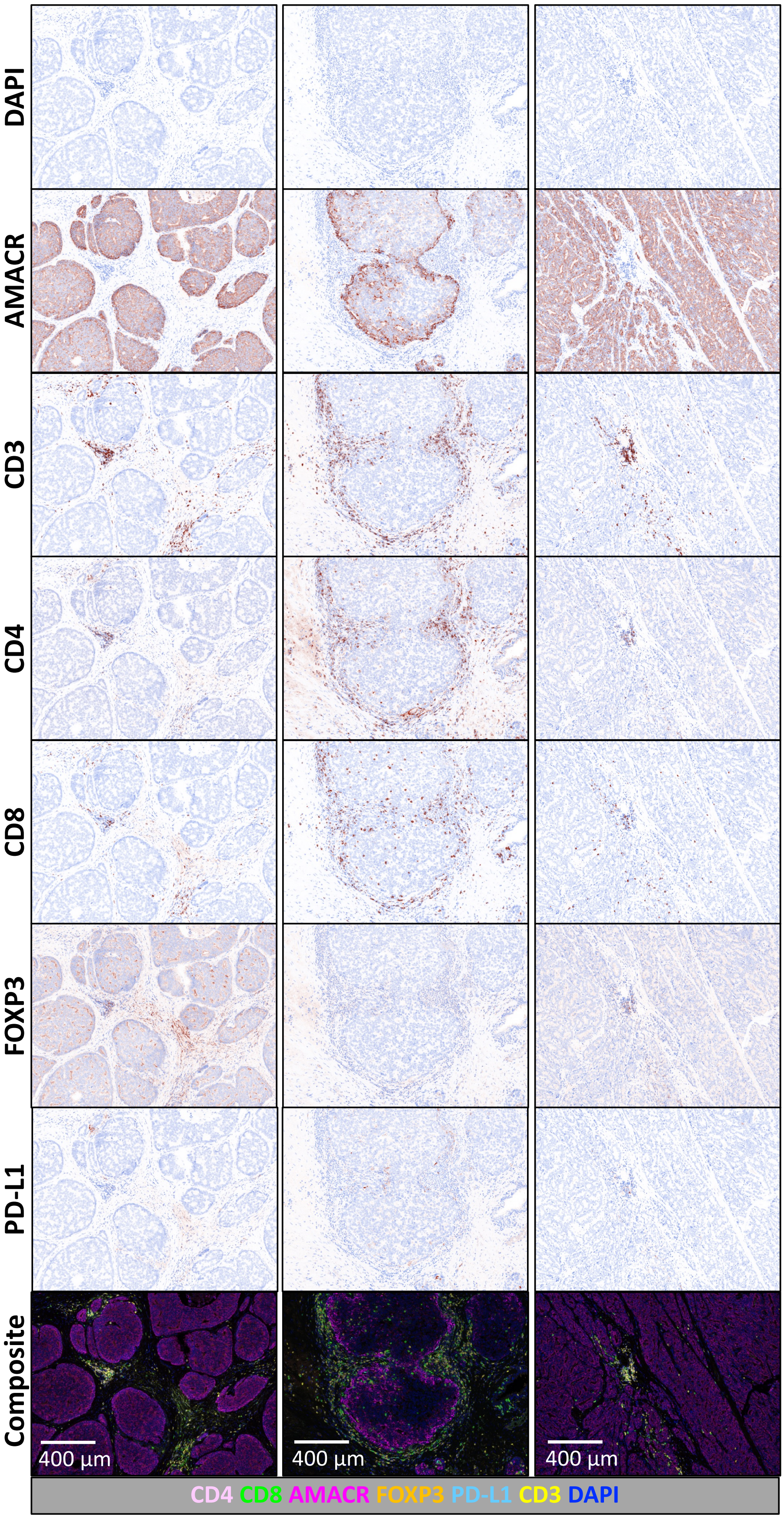

Supplementary Figure 1. Microscopy images from the prostate cancer dataset[1] of each staining and the composite image corresponding to Figure 2b.

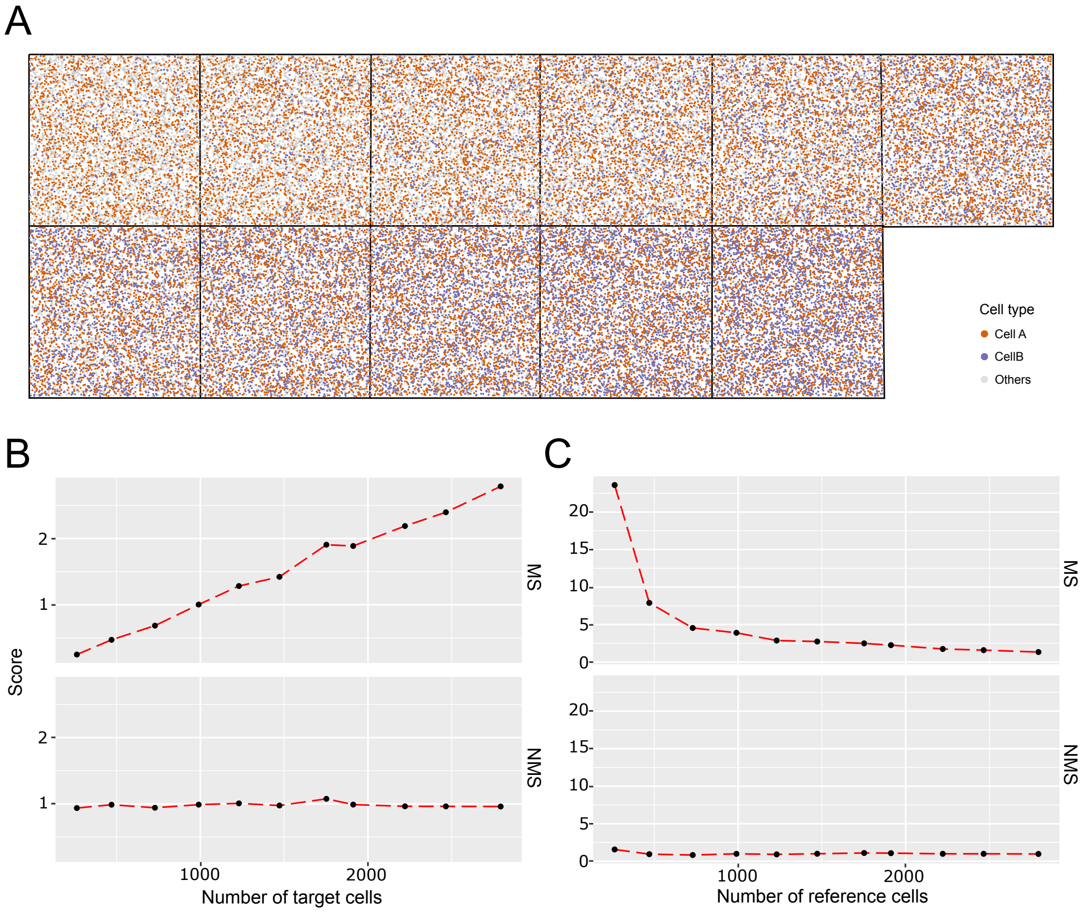

Supplementary Figure 2. Benchmarking the mixing score (MS) and normalized MS (NMS) using simulated images. (A) A set of 11 simulated images. The cell counts of A cells in all images are consistent. The cell count of cells of type B increases from left to right. **(B)** The MS and the NMS calculated with cells of type A as reference cells and cells of type B as target cells. As the number of target cells increase, the MS increases, while the NMS remains consistent. **(C)** The MS and NMS calculated with cells of type B as reference cells and A cells as target cells. As the number of reference cells increase, the MS increases, while the NMS remains consistent. The NMS captures the chances that a target cell type is intermixed among reference cells, given the number of target cells present. In contrast, the MS captures the density of target cells surrounding reference cells, and therefore it is sensitive to the number of target and reference cells. MS: Mixing Score; NMS: Normalized Mixing Score. Source data are provided as a Source Data file.

**Spatial pattern**	**Example image**	**Example cross K plot**	**Percentage of images with positive CKIs**
Obvious immune rings	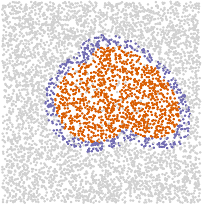	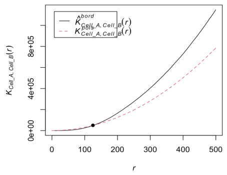	99.8 (of 500 images)
Obvious immune rings; immune cells within tumor and stromal area	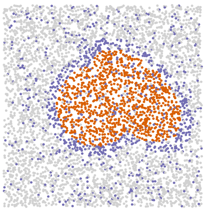	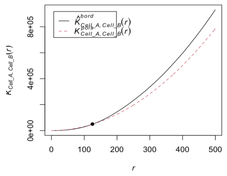	99.4 (of 500 images)
Obvious immune rings; immune cells infiltrating the tumor area	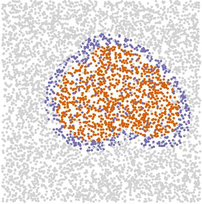	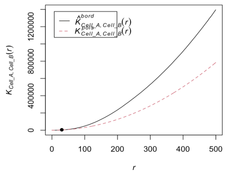	91.4 (of 500 images)
Obvious immune rings but only one layer of immune cells in the rings	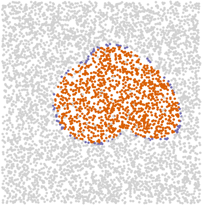	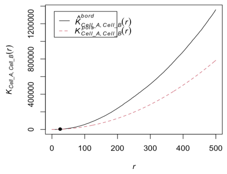	78.6 (of 500 images)
Few immune cells forming not obvious rings around tumor area	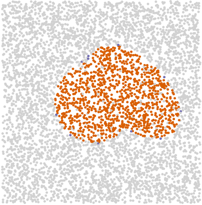	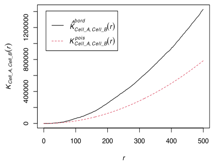	28.2 (of 500 images)
No immune ring, immune cells are randomly located across the image	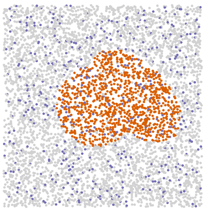	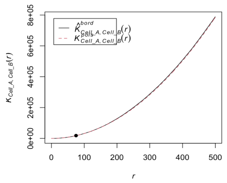	18.8 (of 500 images)
No clear tumor clusters, no immune rings	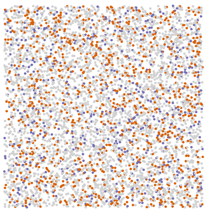	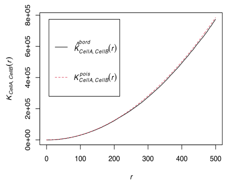	10.1 (of 1000 images)

No immune ring; immune cells are randomly distributed within the tumor area	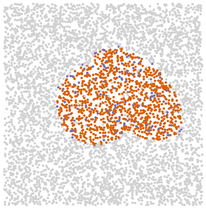	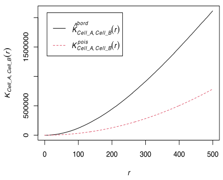	0 (of 500 images)

Supplementary Figure 3. The performance of the CKI across various spatial patterns. We describe in Figure 3 that the CKI captures the presence of immune rings. Here we test the performance of the CKI across distinct spatial patterns. We see that the presence of an immune ring leads to a positive CKI in over 99% of cases. We note that the CKI value can also give a positive value in the absence of immune rings or when there are no clear tumor clusters, we therefore advise the use of multiple complementary spatial metrics to obtain an accurate representation of a pattern. The black dot indicates the point of intersection. CKI: Cross-K Intersection. Source data are provided as a Source Data file.

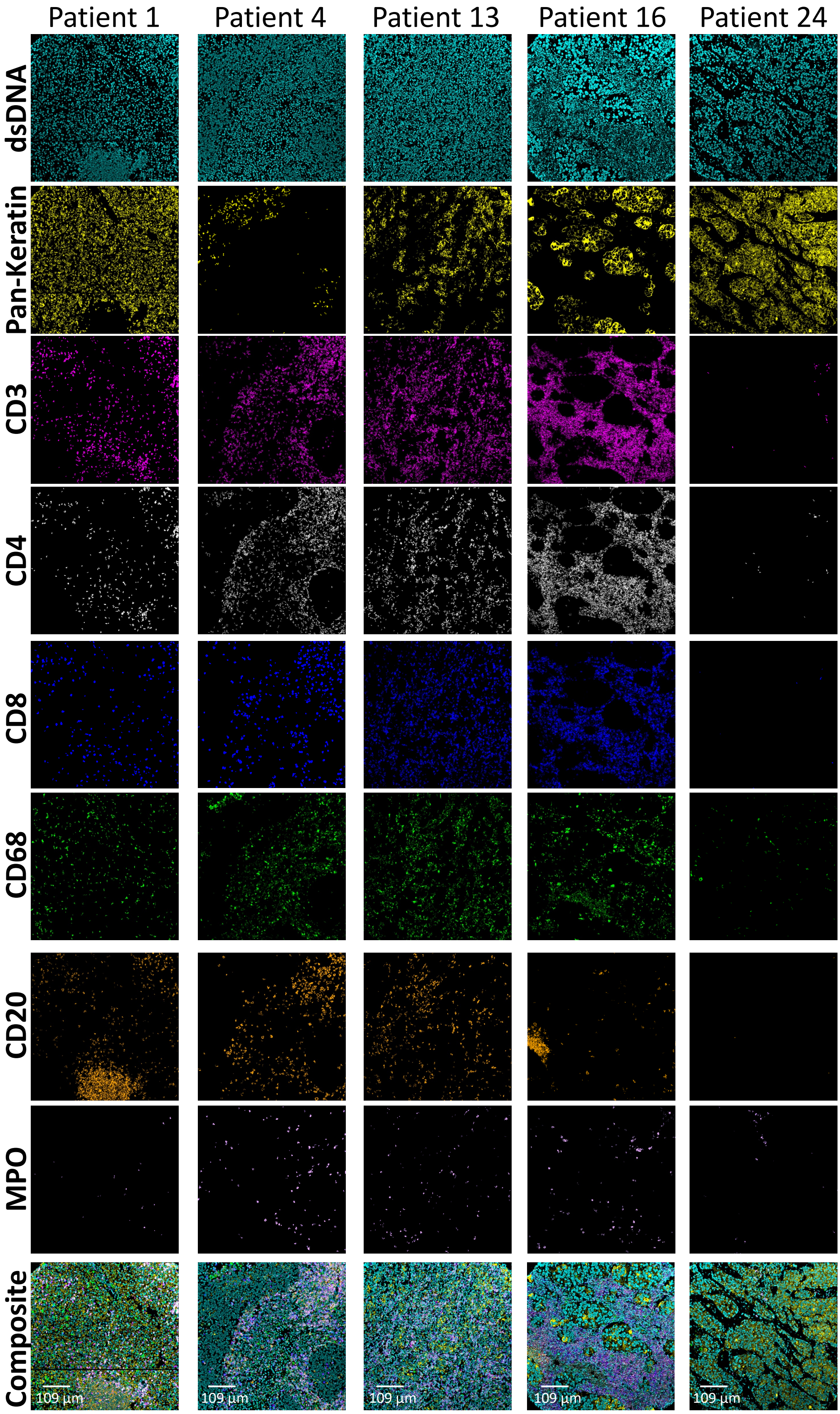

Supplementary Figure 4. Microscopy images of individual stains and the composite image of samples from the MIBI TNBC dataset[2] corresponding to Figures 4d and 5c.

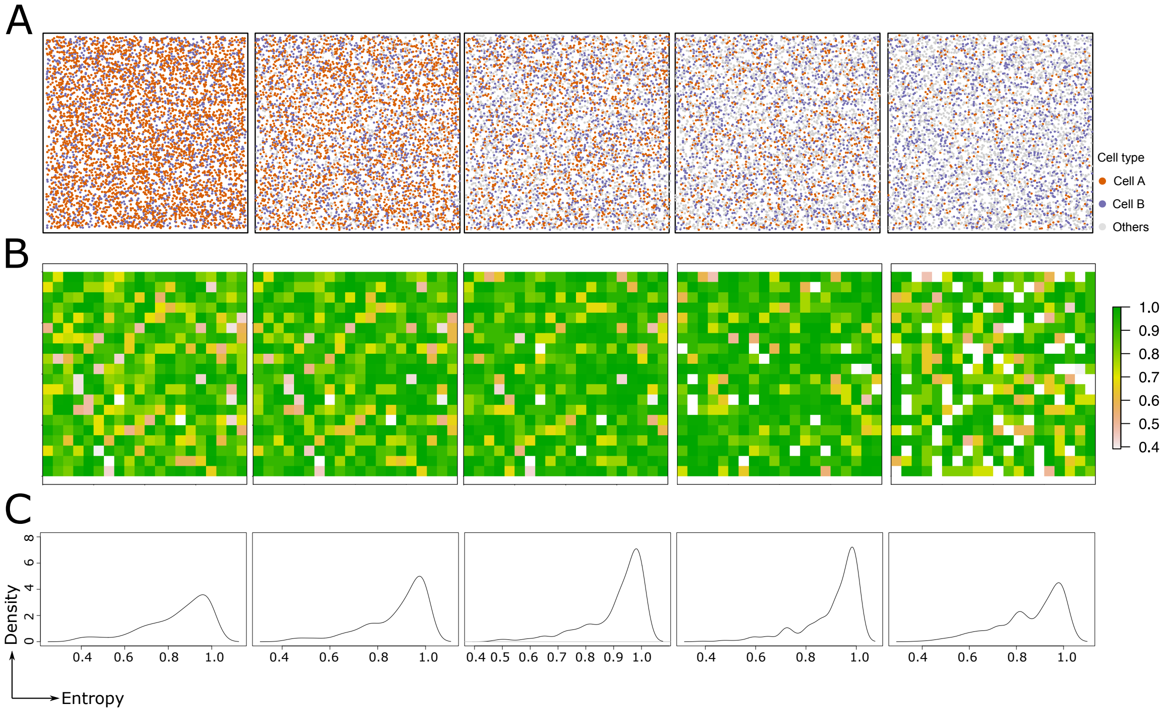

Supplementary Figure 5. Association between entropy values and cell densities. (A) Simulated images with independently located cell types (Cell A and Cell B). From left to right, the ratio of the number of A cells to B cells are 7:3, 2:1, 1:1, 2:3, 1:3. The density of cells of type B remains consistent. **(B)** Entropy grid visualization of the corresponding images in panel (A). Each grid is colored based on its entropy value calculated by the number of A and B cells within each grid. **(C)** Density plots of the entropy values of all grid squares in each plot of panel (B). As the density of the A cells decreases to the density levels of the cells of type B, the localized entropy increases, reaching to a maximum when both densities are equal. After this, further decrease in the density of Cell A causes the entropy to decrease, as the relative proportions of A and B cells in the grid squares tend to become more dissimilar. Source data are provided as a Source Data file.

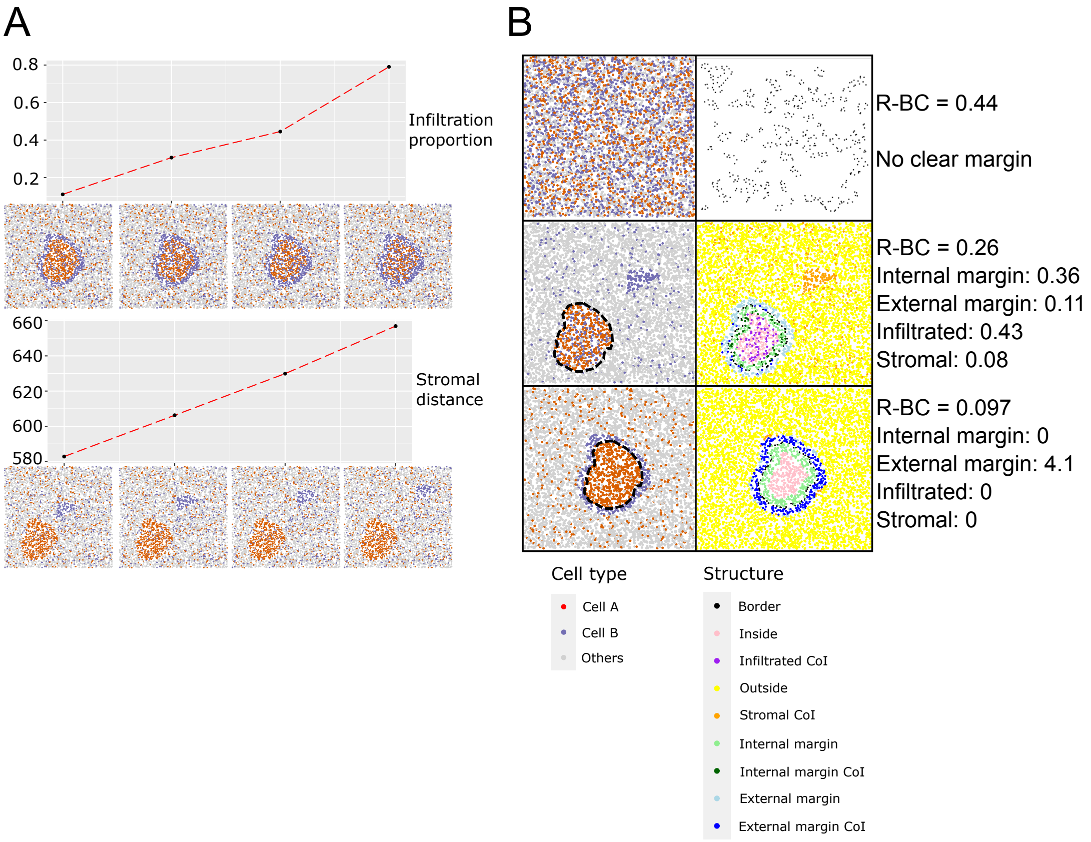

Supplementary Figure 6. Characterizing cell populations relative to a tissue structure margin with images simulated with spaSim. (A) Simulation of images with distinct proportions of infiltrated (top row) and stromal (bottom row) cells of type B. SPIAT was used for automatic margin detection and classification of cell populations based on location. The calculated proportion of infiltrated (top row) and stromal (bottom row) populations matched the expected levels of infiltrated and stromal populations used as input parameters to the simulator. **(B)** Simulated images with various levels of margin clarity showcasing the identification of tissues with clear and unclear margins using the R-BC scores. Simulated images without clear margins had higher R-BC scores (top row), whereas clear margins were associated with lower R-BC scores (middle and bottom row). Proportions shown correspond to the proportion of cells of type B in each location. Blue dotted line around clusters of A cells corresponds to the margin. R-BC: Ratio of Border cell count to Cluster cell count. Source data are provided as a Source Data file.

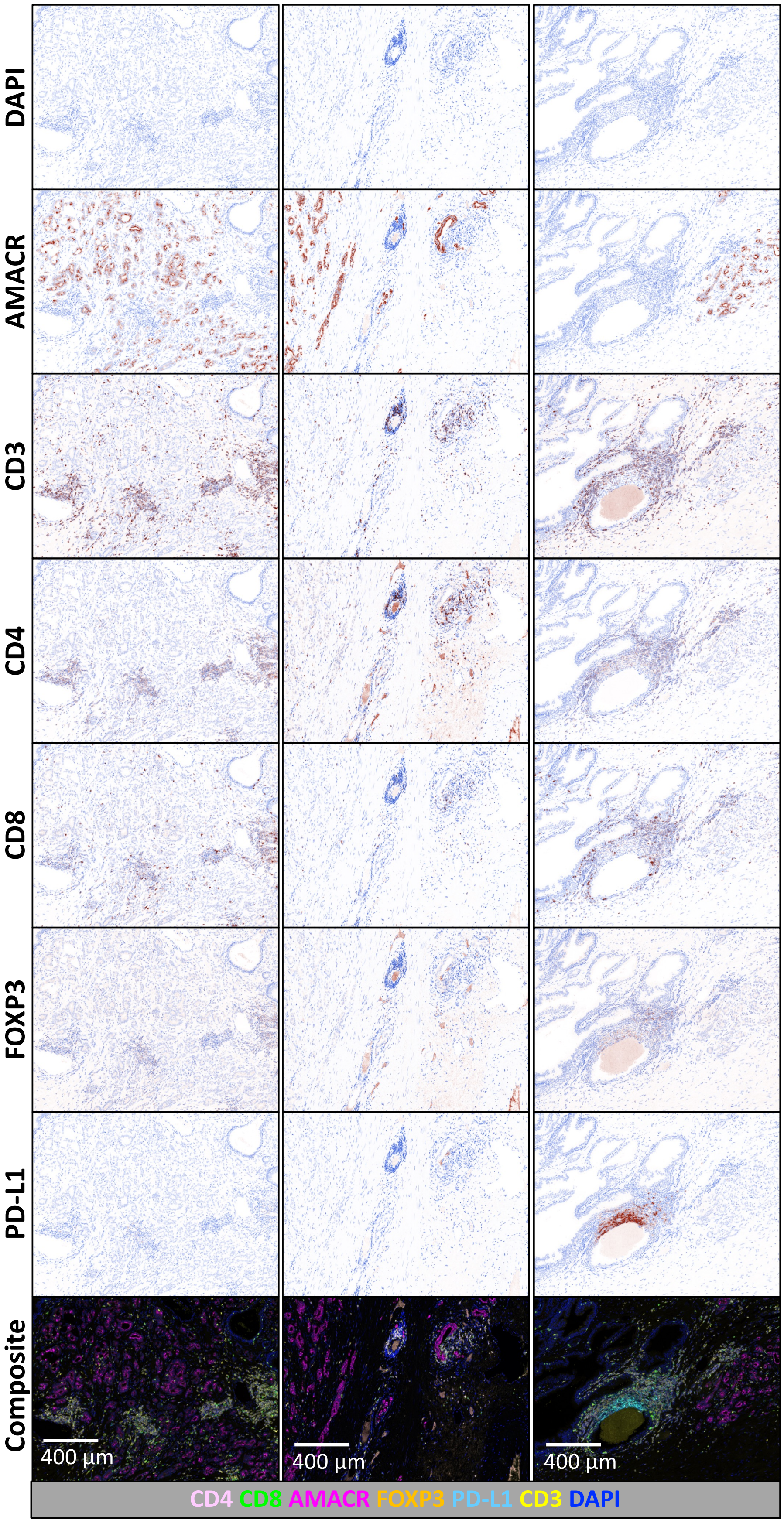

Supplementary Figure 7. Microscopy images from the prostate cancer dataset[1] of each stain and the composite image corresponding to Figure 6b, left.

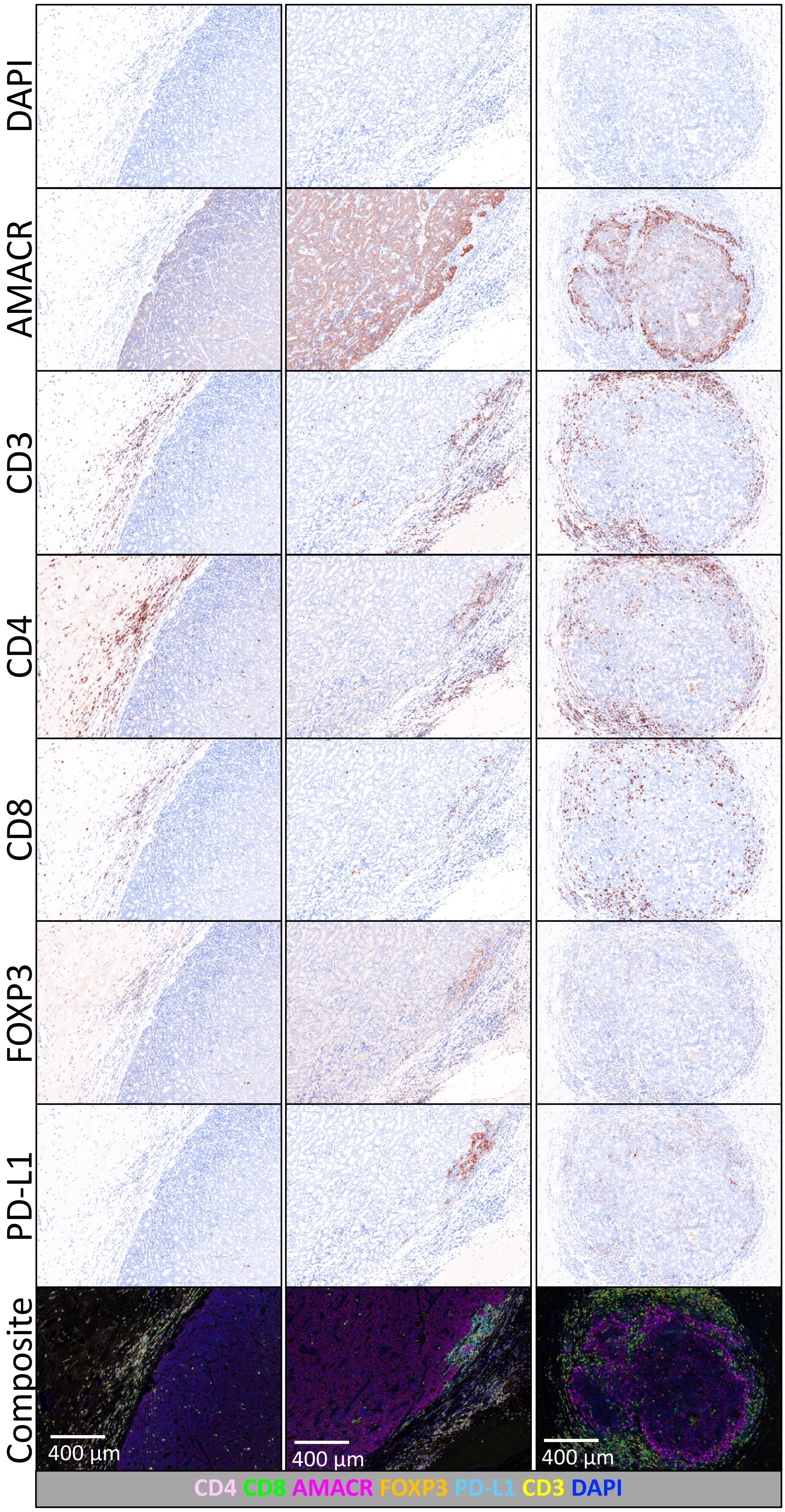

Supplementary Figure 8. Microscopy images from the prostate cancer dataset[1] of each stain and the composite image corresponding to Figure 6b, right.

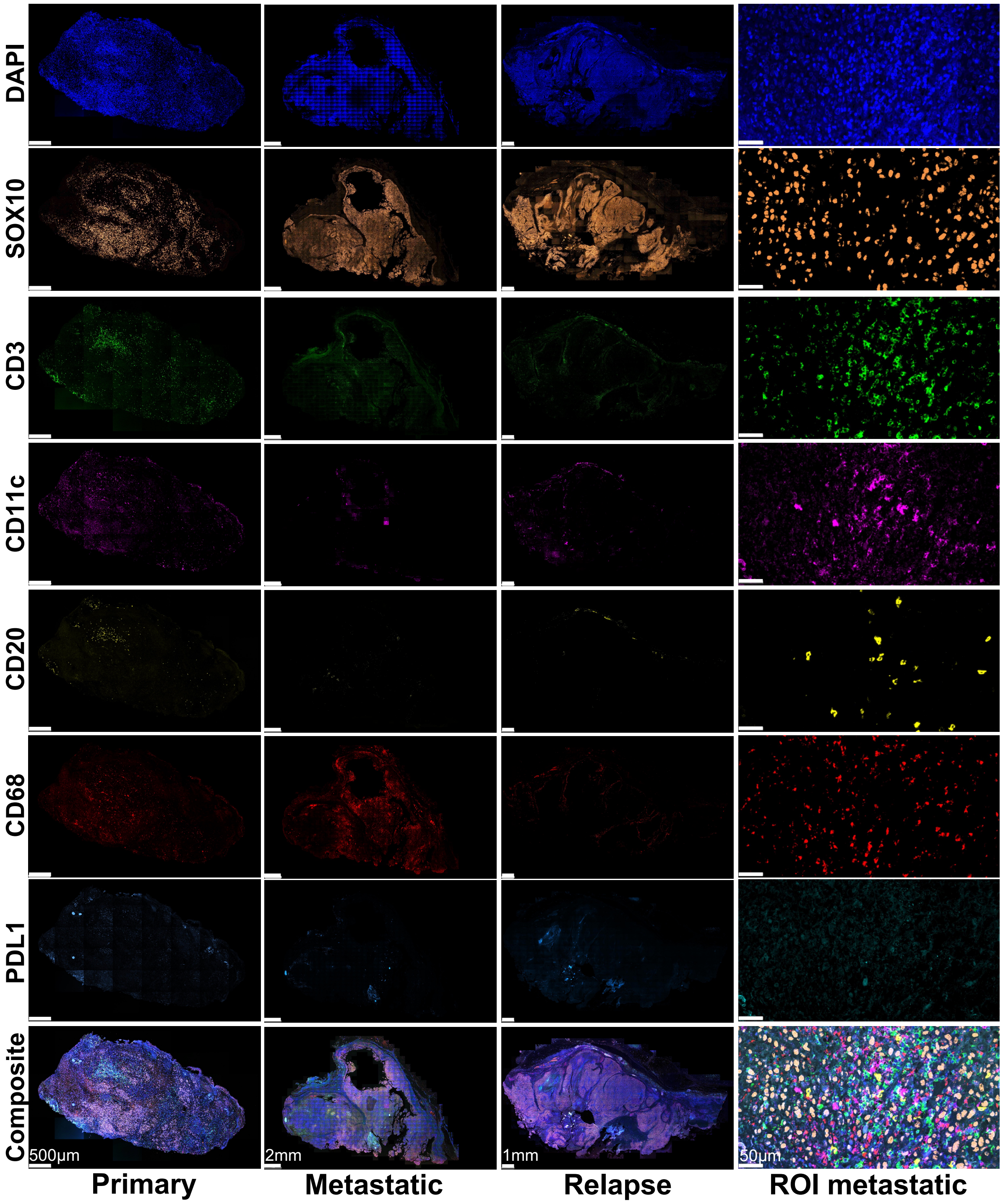

Supplementary Figure 9. Microscopy images from the melanoma dataset[3] of individual stains and the composite image corresponding to Figure 6c. A closeup ROI of the metastatic sample is shown.

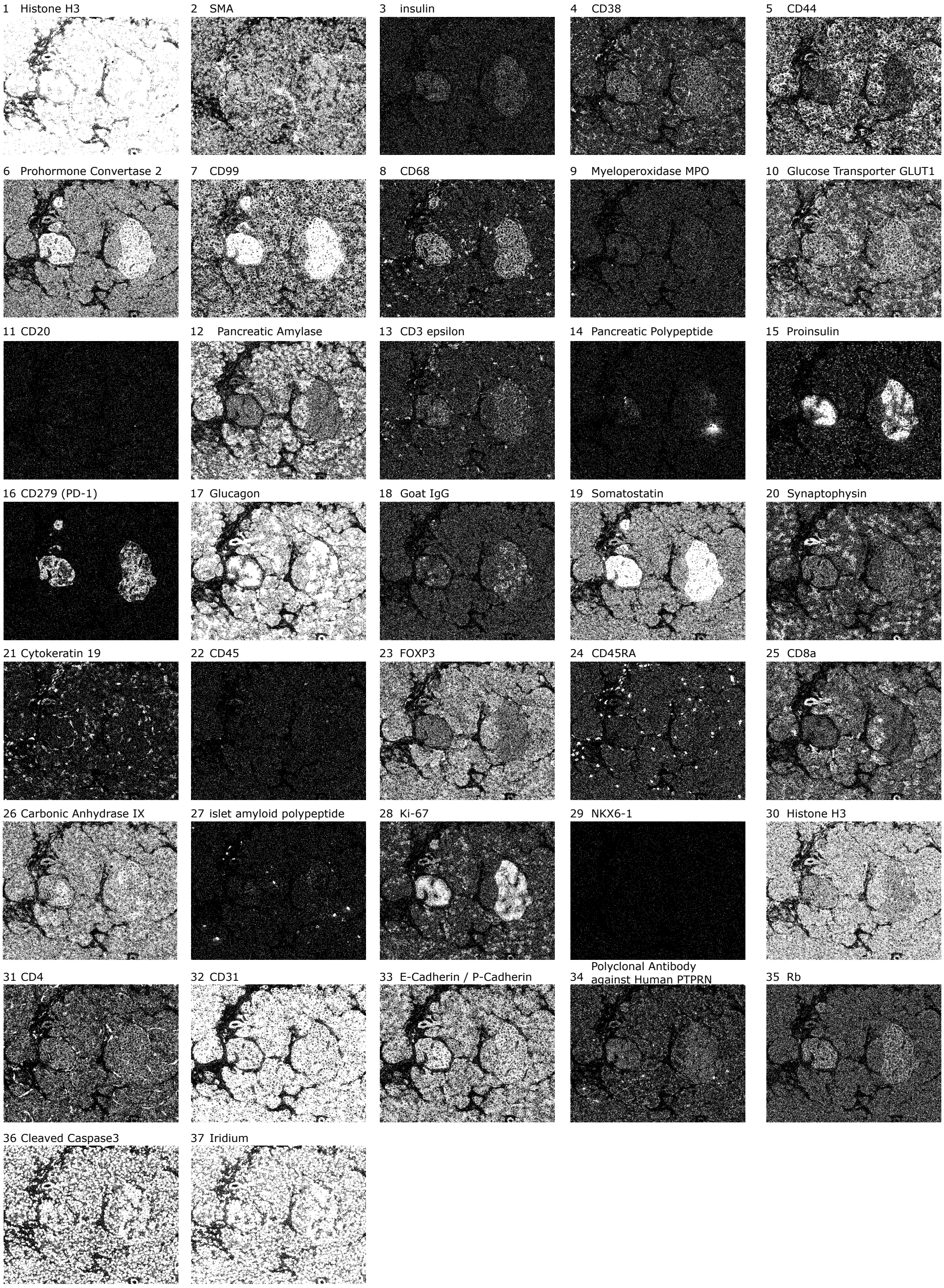

Supplementary Figure 10. Single-channel images of individual markers corresponding to Figure 6d from the diabetes IMC dataset[4].

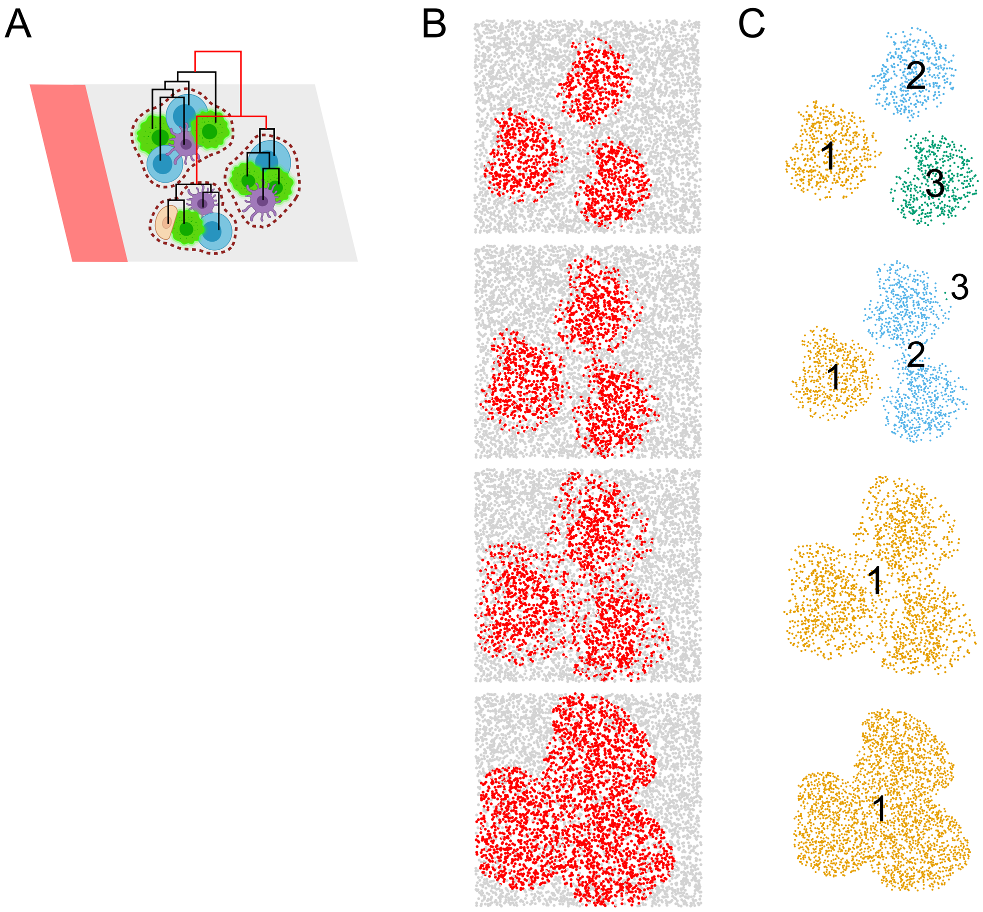

Supplementary Figure 11. Hierarchical clustering algorithm in SPIAT. (A) Diagram of the identification of neighborhoods with the hierarchical clustering algorithm. **(B)** Simulations of clusters with spaSim and **(C)** their identification using our hierarchical clustering algorithm. Less defined clusters are less likely to be separated. Source data are provided as a Source Data file. Created with BioRender.com.

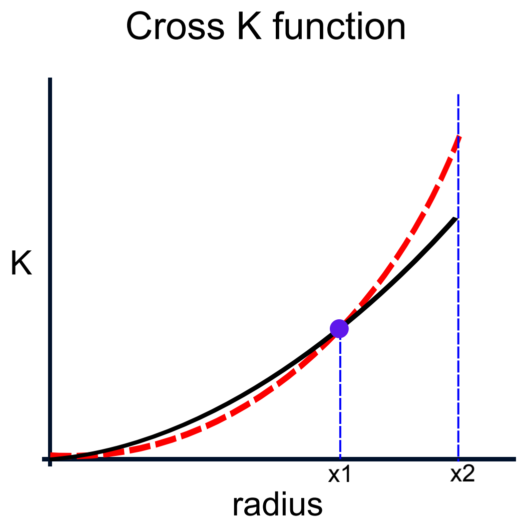

Supplementary Figure 12. An illustration of the CKI metric. The horizontal axis is the radii used by the cross K function between two cell types. The dashed curve is the expected cross K function estimated with a Poisson. The black curve is the observed cross K function. The solid point represents the intersection of the observed and expected cross K curves. r: Radius, K: cross K function value.

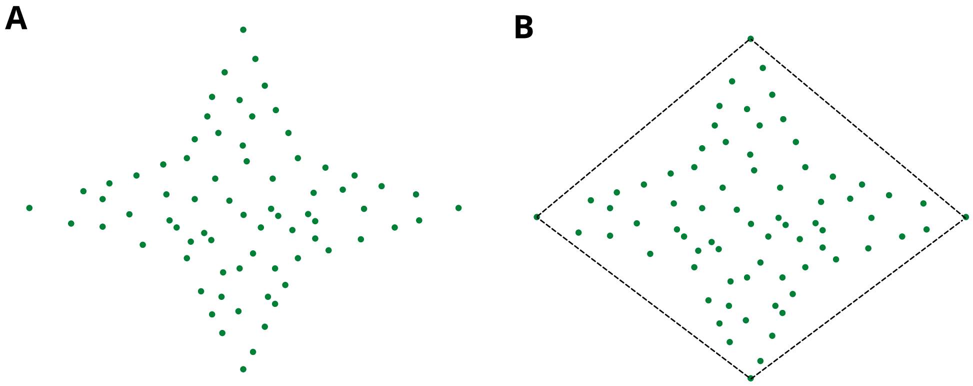

Supplementary Figure 13. An illustration of convex hull. (A) A point pattern of an arbitrary shape. **(B)** Black lines outline the convex hull of the point pattern.

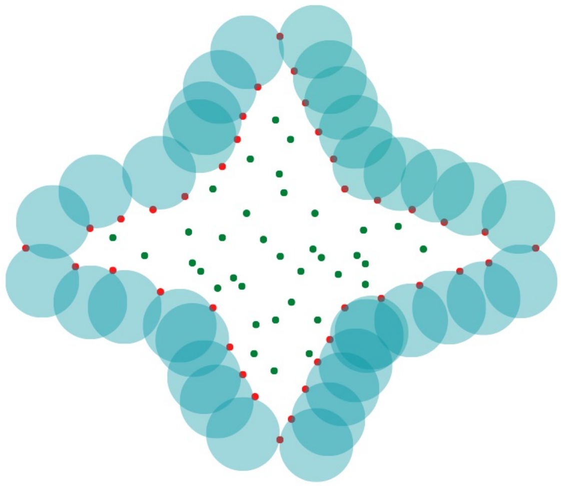

Supplementary Figure 14. An illustration of the alpha hull. The blue circles are of the same radius, alpha. The red points are the ones on the alpha hull representing the bordering cells of the point pattern. These represent the bordering cells.

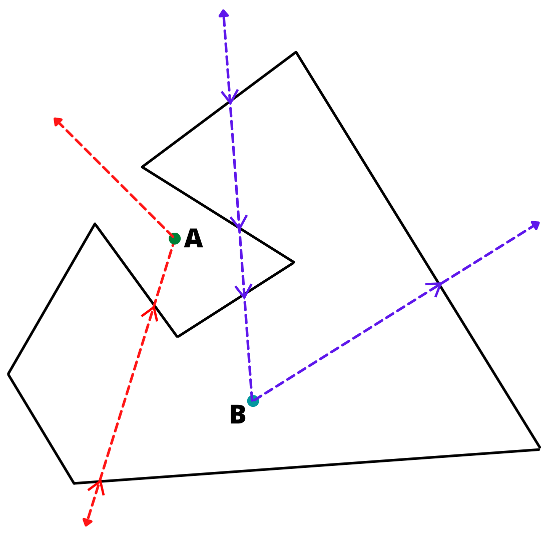

Supplementary Figure 15. An illustration of the ray crossing algorithm. Point A is outside of the polygon; the rays emitting from A cross the polygon 0 or 2 (even) times. Point B is inside of the polygon; the rays emitting from B cross the polygon 1 or 3 (odd) times.

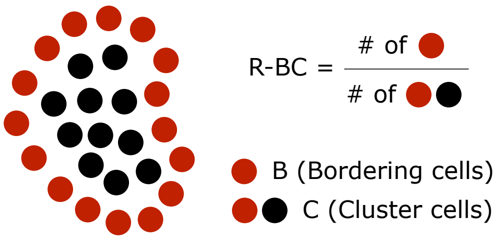

Supplementary Figure 16. An illustration of the R-BC metric. R-BC: Ratio of Border cell count to Cluster cell count.

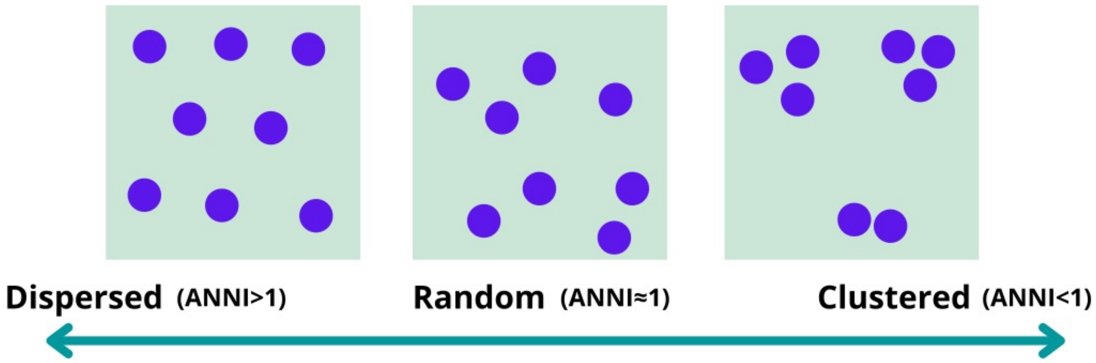

Supplementary Figure 17. An illustration of ANNI. ANNI: Average Nearest Neighbor Index.

Supplementary Table I. Comparison of the capabilities of SPIAT and other spatial analysis tools

Tool	SIMPLI[5]	HistoCAT[6]	Cytomapper[7]	Imcyto[8]	Giotto[9]	ImaCytE[10]	Immunocluster[11]	CytoMAP[12]	Squidpy[13]	SPIAT
Environment	Nextflow	Matlab	R	Nextflow	R	MATLAB	R	MATLAB	Python	R
QC	Y	N	N	N	N	Y	Y	N	N	Y
Distance-based analysis	Y	Y	N	Y	N	N	N	Y	Y	Y
Cell colocalization metrics	N	N	N	N	N	1 method	N	N	1 method	7 methods
Automated margin detection	N	N	N	N	N	N	N	N	N	Y
Defining de novo cellular neighbourhoods based on cell identities	1 method	2 methods	N	1 method	N/A	1 method	N	1 method	1 method	3 methods
Testing the presence of pre-defined neighbourhoods	N	N	N	N	N	N	N	N	1 method	1 method
Measuring the spatial heterogeneity of cell colocalization	N	N	N	N	N	N	N	N	N	Y
Colocalization independent of thresholding	N	N	N	N	N	N	N	N	N	Y
Raw image processing	Y	Y	N	Y	N/A	N	N	N	Y	N

Cell phenotyping	Y	Y	Y	Y	Y	Y	Y	N	Y	Y
Benchmarking	N	N	N	N	N	N	N	N	N	Y (spaSim)

Supplementary Table II. Comparison between spatstat and spaSim simulators

	**spatstat**	**spaSim**
**Basis**	Point process (based on statistical distributions)	Point process + deterministic features (e.g. geometric shapes)
**Multitype**	Only basic patterns	Both basic and complex tissue patterns
**Multiple structures per image**	No	Yes
**Clustering**	Not representative of tissues	Representative of tissues
**Simulates tumor and stromal clusters**	No	Yes
**Simulates immune rings**	No	Yes
**Simulates immune infiltration**	No	Yes
**Allows generating a range of images based on fluctuations of a parameter**	No	Yes

Supplementary Table III. Summary of benchmarking results of colocalization metrics

	**Can distinguish stroma and infiltrated immune cells**	**Can measure degree of immune infiltration**	**Can measure distance to stromal immune**	**Can identify immune ring formation**
**APD**	Y	N	Y	N
**AMD**	Y	N	Y	N
**NMS**	Y	N	N	N
**Cross-K AUC**	Y	N	N	N
**CIN**	Y	Y	N	N
**MS**	Y	Y	N	N
**CKI**	N	N	N	Y

Note N1

To showcase the power of SPIAT in characterizing the spatial distribution of cells in tissues, we include results from a primary prostate cancer tissue section profiled with 7-color OPAL multiplex IHC stained for CD3, CD4, CD8, FoxP3, PD-L1, AMACR and DAPI (Figure N1.1).

As a first step in our image analysis, we phenotyped cells with SPIAT (Figure N1.1A). In this process, we defined which cells were positive for each profiled marker based on the density plot of marker intensities (Figure N1.1B). The phenotype of cells closely matched the reference IHC stains (Figure N1.1A). As expected, the intensity of the CD8 marker in individual cells matched their assigned phenotype (Figure N1.1C). SPIAT also implemented dimensionality reduction plots, including UMAP and tSNE plots based on marker intensities. Our interactive implementation allows users to identify potentially misclassified cells and exclude them from further downstream analysis. A tSNE plot based on marker intensities revealed 4 distinct clusters, each corresponding to a distinct population of cells (Figure N1.1D). Based on marker combinations, we then defined 'cell types' (e.g. $CD3^{+}CD8^{+}$ cells correspond to cytotoxic T cells), exclude undefined cells and calculate the percentage of each within the image (Figure N1.1E).

Next, we aimed to obtain a visual understanding of the spatial distribution of cells after phenotyping. SPIAT provides multiple options for visualization, including plots by cell type and individual marker intensities (Figure N1.1F). The visualization options in SPIAT allowed a quick visual assessment of patterns of immune infiltration, with multiple aggregates of immune cells close to some tumor areas, and regions where markers co-occur at close locations.

To quantify this, SPIAT can calculate the average minimum distance between cell types which can be visualized as a heatmap (Figure N1.1G). SPIAT allows extracting the minimum distances for each cell type, which allows a comparison of distributions (Figure N1.1H). For example, the distance of cytotoxic T cells to $PD\text{-}L1^{+}$ cells is larger than to helper T cells (two-sided Wilcoxon test p-value $< 10^{-6}$), suggesting that they occupy distinct cellular environments.

In summary, SPIAT allows visualization and basic spatial analysis, including calculation of cell percentages and distances between cells, enabling the identification of interacting cell populations.

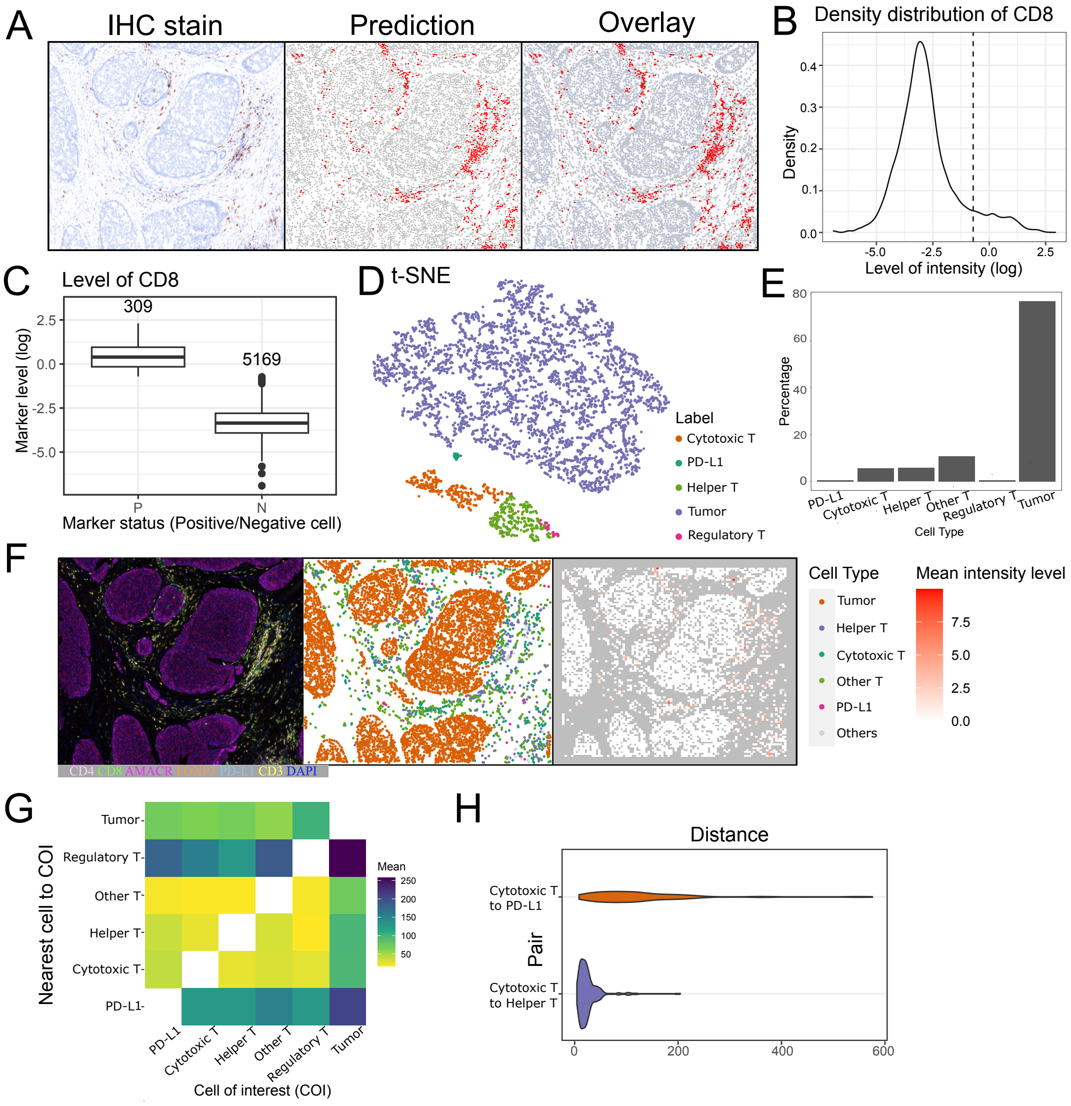

Figure N1.1. Standard analysis performed by SPIAT. (A) Results obtained with automated phenotyping with SPIAT. IHC staining for CD8 (left), cells predicted to be positive to CD8 (red dots) (middle), overlay between the IHC staining and prediction showing overlap (right). **(B)** Density distribution of the intensity of the CD8 marker. The vertical dotted line corresponds to the threshold identified by SPIAT. Cells to the right of the dotted line were classified as positive for the marker. **(C)** Intensity of the CD8 marker in cells phenotyped as positive and negative. The numbers at the top of the boxplots corresponds to the number of cells. **(D)** A tSNE plot built using marker intensities. Each dot corresponds to a cell. Cells were colored by cell type. **(E)** Percentages of PD-L1+, cytotoxic, helper T cells and tumor cells in the image. **(F)** Visualization of marker intensities. Image obtained with the inForm software showing the distinct markers (left), cell type plot showing the distinct phenotyped cells (middle), and marker levels of CD8 (right). **(G)** Average minimum distances between cells (µm) calculated and plotted with SPIAT. **(H)** Minimum distances between cytotoxic T cells and PD-L1+ cells, and cytotoxic and helper T cells in SPIAT. Cytotoxic T cells are further from PD-L1+ cells than to helper T cells (two-sided Wilcoxon test p-value $1.60\text{x}10^{-72}$). In boxplots, the center line corresponds to the median, the box limits correspond to the first and third quartiles (the 25th and 75th percentiles). The upper and lower whiskers extend to the maximum or minimum value within 1.5 times the interquartile range, respectively. Data points beyond the whiskers are plotted individually. IHC: Immunohistochemistry; t-SNE: t-distributed Stochastic Neighbor Embedding; T_cyto: Cytotoxic T cell; T_helper: Helper T cell; Treg: Regulatory T cell; T_other: CD3+ cells; Dist: Distance; COI: Cell Of Interest. Source data are provided as a Source Data file.

Note N2

Although one of the utilities of the R package spatstat is to simulate point data in 2D space (using spatstat.random v3.0-1), our package spaSim implements a different approach that results in several advantages over spatstat. spaSim's ability to simulate patterns representative of structures and clusters found in tissue sections is based on its approach of using geometric shapes to present cell spatial patterns on top of background cells generated using a point pattern process (see Methods), while spatstat simulates point clusters by statistical models. A summary of the main differences can be found in Table SII.

Randomly distributed mixed cell types can be achieved with both spatstat and spaSim (Figure N2.1), with some key differences. spaSim implements the Hardcore process simulation from spatstat and an intuitive evenly spaced distribution model to simulate background cells in a 2D space. Next, we use random number sampling to assign a cell type to each background cell based on proportions specified by the user. This simulation requires the specification of the desired cell types, cell proportions, window size, total cell number, and the minimum distance between any two cells.

In contrast, simulating mixed cell types by spatstat can be achieved by using the rmpoispp() function which simulates a multitype Poisson process. The simulation needs the specification of the intensities of the Poisson processes, the window size and the cell types. The total cell count and the proportion of the cell types are controlled by intensities.

The images simulated by spatstat multitype Poisson process (Figure N2.1A) and by spaSim mixed background cell simulation (Figure N2.1B) are similar and the proportions of cell types generated are both close to the proportions specified. However, two differences emerged in the process of simulations. First, spatstat requires users to specify the intensity of each cell type to indirectly obtain a certain number of each cell type and total cells, which is not intuitive. In spaSim this is done easily by directly specifying the total cell counts and the proportion of each cell type. Second, spaSim uses a Hardcore process or an evenly spaced distribution model to simulate the cells that result in cells separated from each other. This is more realistic for a simulator of tissue images as cells take up volume and therefore there is a certain distance between any two cells.

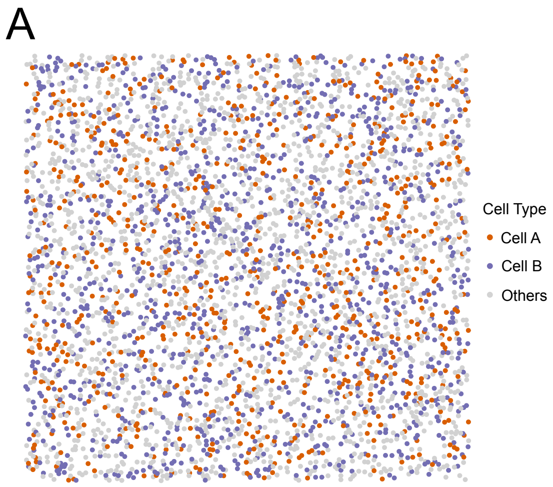

Figure N2.1. Simulated images by spatstat package (left) and by spaSim package (right). The desired cell count is 4000, where the ratio of Cell A, Cell B and other cells is 2:3:5. The image generated by spatstat has in total 3979 cells generated with 818, 1169, and 1992 cells for each type. The image generated by spaSim has in total 4000 cells generated with 779, 1209, and 2012 cells for each type.

The largest differences between spatstat and spaSim comes when simulating more complex images, with combinations of cell types following different distributions, and these often relative to each other. To showcase this difference, we aimed to simulate a tumor area based on what is routinely observed in prostate cancer tissue (Figure N2.2A, Figure N2.2B shows tumor cells) with spatstat (Figure N2.2C,D) and spaSim (Figure N2.2E,F). There are several cluster models available in spatstat to achieve this. Here we tested both the Thomas and Cauchy cluster models. First, we fit the models to the real image data. The parameters estimated include kappa (the intensity of the Poisson process of cluster centers), scale (the standard deviation of random displacement), and mu (the mean cluster size, which is the cell counts in each cluster). Simulations are then conducted by rThomas() and rCauchy() functions by applying the estimated parameters (Figure N2.2C,D). Others have previously used the Thomas process to simulate cell clusters[14]. We can observe that the images simulated with spatstat have multiple clusters (as their number cannot be specified), tumor cells are less aggregated than expected, with no clear tumor area. Generally, spatstat generates simulations where clusters are dense in the center, and less compact in the periphery, which is generally not a pattern observed in tumor tissues. spatstat cannot simulate the relationship between tumor and immune cells.

In contrast, spaSim was able to simulate an image that largely captures the distribution of tumor cells present in the original image (Figure N2.2E). Furthermore, spaSim can include layers of as many different cell types desired, making it possible to generate simulated images that capture realistic patterns of tumor-immune interactions (Figure N2.2F).

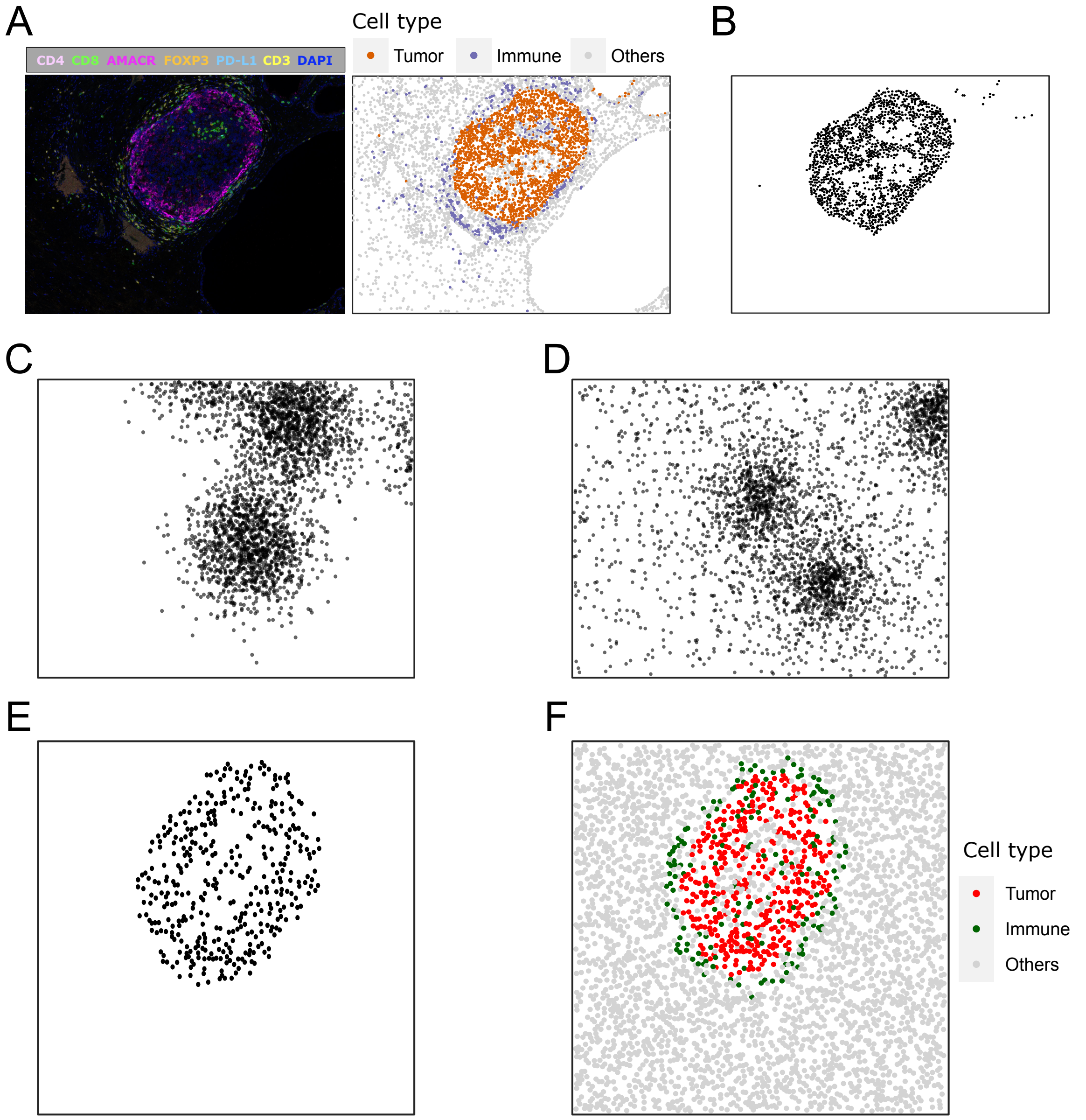

Figure N2.2. Simulating cell clusters based on a real image. (A) Example prostate cancer composite microscopy image. **(B)** Plot showing the locations of the tumor cells in the real image. **(C)** A simulated image by rThomas() function from spatstat. **(D)** A simulated image by rCauchy() function from spatstat. **(E)** Locations of tumor cells stimulated by spaSim. The tumor cells form a cluster, similar to (A). **(F)** A simulated image by spaSim colored by cell types. The simulation includes background cells, tumor cluster cells and immune cells. The immune cells form a ring, also similar to the original image (A). This type of multitype image with cells having different distributions, but being relative to each other, is not possible with spatstat. Source data are provided as a Source Data file.

Note N3

Here we aim to validate the simulation process of spaSim. The purpose of spaSim is to test spatial metrics in a controlled environment across different ranges of spatial patterns to understand their behavior in different settings, as we have done in Figures 3, 4, 5 and 6. Fundamental to this is spaSim's ability to capture and replicate specific spatial properties of individual images, such as cell co-localization, clustering and cell proportions. We have therefore focused our validation on the ability of our simulations to reproduce spatial properties of real images, namely (1) cell proportions, (2) co-localization and (3) clustering metrics. Table N3.1 includes a breakdown of specific metrics to be validated.

Table N3.1. Spatial metrics to be validated with spaSim

Spatial property	Symbol	Description	SPIAT function to calculate metrics
Cell composition	$PT_{cell\ types}$	Proportion of individual cell types (beta, non-beta, endothelial, immune and other) in the whole image	calculate_cell_proportions()
Co-localization	APD	Average pairwise distance between islet (beta and non-beta) cells and endothelial cells	calculate_pairwise_distances_between_celltypes(); calculate_summary_distances_between_celltypes()
Co-localization	AMD	Average minimum distance between islet (beta and non-beta) cells and endothelial cells	calculate_minimum_distances_between_celltypes(); calculate_summary_distances_between_celltypes()
Co-localization	MS	Mixing score between islet and endothelial cells	mixing_score_summary()
Co-localization	NMS	Normalized mixing score between islet and endothelial cells	mixing_score_summary()
Co-localization	CIN	Cells in Neighbourhood between islet and endothelial cells	average_percentage_of_cells_within_radius()
Co-localization	AUC	AUC scores between islet and endothelial cells	calculate_cross_functions(); AUC_of_cross_function()
Clustering	R-BC	Ratio of Bordering to Clustered cells	R_BC()
Clustering	$N_{derived\text{-}islet\text{-}cells}$	Number of cells within islets	identify_bordering_cells()

To achieve this, we simulated images based on images from a reference dataset and then determined how the spatial metrics from the simulated images compared with those of the reference dataset. We used as a reference dataset the 845 real images from the diabetes dataset generated by IMC[4] due to its large number of images. In this dataset, each image contained at least one islet with islet cells (beta and non-beta cells), and immune and endothelial cells primarily found in the stromal area. Further details about this dataset can be found under the "Datasets" header in the Methods section. Basic metrics were extracted from

this dataset using SPIAT (Table N3.2). Finally, using spaSim we generated a 'paired' simulated image for each of the images of the reference dataset based on the features that were extracted.

Table N3.2. Features extracted and estimated from real images

Feature type	Symbol	Description	SPIAT function for the feature extraction
Extracted from real image	N_{cells}	Total number of cells per image	-
	$P_{celltypes-islets}$	Proportion of islet cell types (beta and non-beta) within islets	Functions from tissue structure identification:<br>identify_bordering_cells()<br>define_structure()<br>calculate_proportions_of_cells_in_structure()
	$P_{celltypes-stroma}$	Proportions of stromal area cell types (immune, endothelial and other) in the stromal area	As above
	N_{islets}	Number of islets	identify_bordering_cells()
	$N_{islets-cells}$	Number of cells within the islets	identify_bordering_cells()
Estimated from extracted variables	D	Global mean of the density of the cells in the reference dataset	-
	J	Measure of cell jittering from their hexagon vertices (see Section 3.2 for details)	-
	S	Size of the image. Estimated from D and N_{cells}	-
	d	Diameter of islets	-

The simulation procedure

First, the extracted features from the real images were used to simulate the background cells, the islets and the stroma (Table N3.3):

Table N3.3. Features used as input to spaSim

Symbol	Description
N_{cells}	As above
S	As above
J	As above
N_{islets}	Number of clusters
$N_{islet-cells}$	Number of cells within the islets
$P_{celltypes-stroma}$	Proportion of immune, endothelial and other cells in the stromal area
$P_{celltypes-islets}$	Proportion of beta and non-beta cells within the islets

Given that spaSim also requires parameters for cell jittering (*J*) and the diameter of clusters (*d*) but these were not easily extracted from the real images, these were estimated based on the extracted values:

- Since the reference dataset had a variable number of image sizes and numbers of cells, while the density of cells remained roughly the same, we adopted the mean of the densities (*D)* of real images as a fixed value for the density of each simulated image. We then extracted the number of cells of each real image, and calculated the image size S as S = number of cells/density. Figure N3.1 shows the distribution of the estimated height and width of images compared to those of the real images, indicating that our procedure to derive the image sizes was sensible. The diameter of clusters *d* was then calculated from the area of clusters that was estimated from multiplying *D and the* $N_{islet-cells}$ extracted from the image.

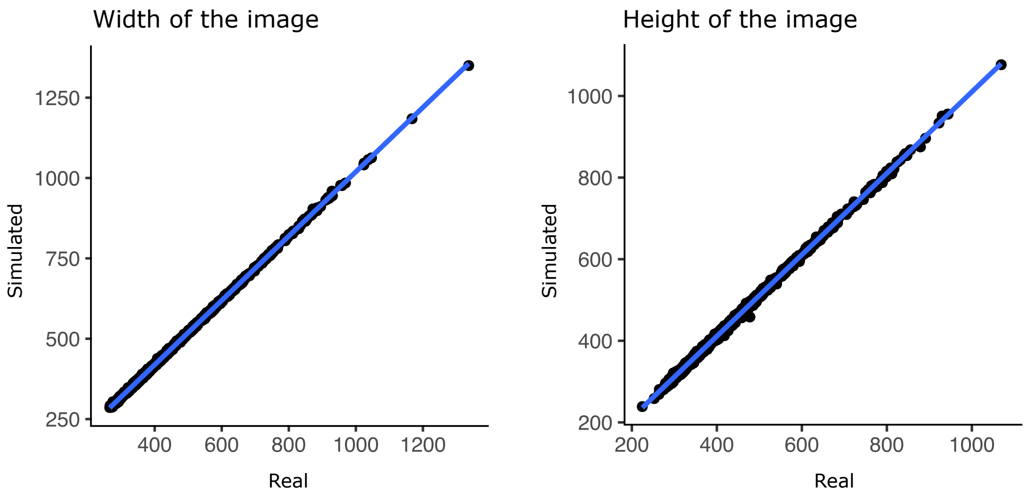

Figure N3.1. Estimated Height and width of estimates used as parameters for simulations. The regression lines that represent the relationship between the simulated and real images are plotted. Source data are provided as a Source Data file.

- To estimate J, we simulated a set of 15 images with jitter values that ranged from 0.25 to 0.32 by a step of 0.005 for each of the reference images. We then calculated the difference between the expected and observed average minimum distance between cells. We selected J to be the jitter value that results in the most similar expected and observed average minimum distance.

Having all the parameters required to carry out the simulations with spaSim, we first simulated background cells using an evenly spaced distribution model using N_{cells}(total number of cells per image), S (size of image) and J (jitter to apply to each cell). See the "Tissue spatial simulator (spaSim)" header under the Methods section of the main paper for the details of the background simulation models. The proportions of cell types in the stromal area were simulated according to the proportions from $P_{celltypes-stroma}$ by assigning a random cell type to each background cell. Note that the locations of one cell type were independent of the locations of another cell type.

To simulate islets, we created N_{islets} islet clusters, each with $N_{islet-cells}$ cells (includes both islet and non-islet cells) within each cluster according to the proportions from $P_{celltypes-islets}$. Clusters were assumed to be circles, and the cluster radii were determined by D, the global density of cells in the diabetes images. We assumed the locations of the islets were in the center of the images. If there was one islet, the center location was the center of the image, if there were more than one islet, the center of the islets was the center of the image and the islets were spaced around the center.

Comparing the metric distribution of the real and simulated paired images

The simulated images visually captured the properties of the real images (Figure N3.2).

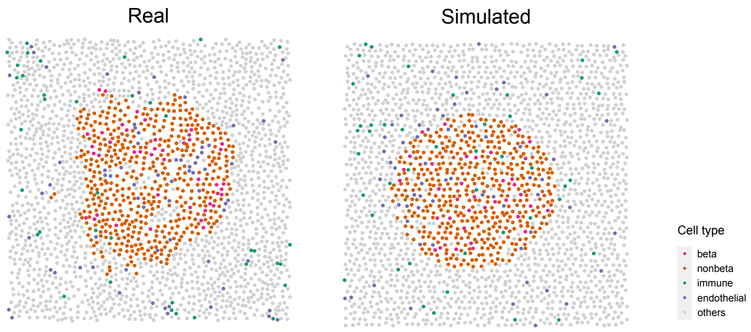

Figure N3.2. Example of a real and its paired simulated image generated by spaSim. Source data are provided as a Source Data file.

To quantitatively compare these similarities, first, we compared the proportion of cell types across the real and simulated images. Note that we segmented the real images into islet and stromal regions, and then derived the proportion of beta and non-beta cell types from the cells that were specifically in the islet area, and derived the proportion of immune, endothelial and other cells from the cells that were specifically in the stromal area. These were then used as input to spaSim. Here we show the comparison of the proportion of cell types across the entire image (Figure N3.3). There is a strong association between the cell proportion values in all cases, with R^2 values between 0.89 and 0.97.

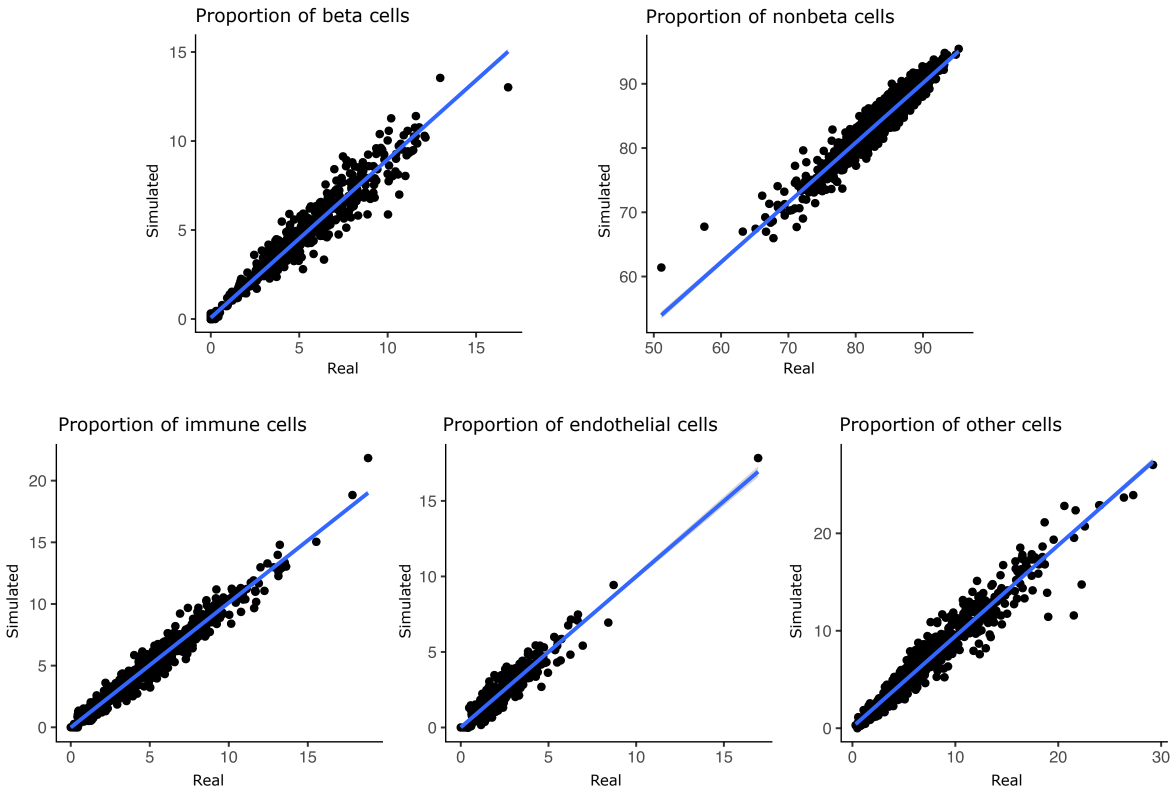

Figure N3.3. Scatterplot of the proportion of cell types across the entire image in real and simulated images. R^2 for beta cells = 0.97; for non-beta cells = 0.94; for immune = 0.97; for endothelial cells = 0.89; for other cells = 0.92. The error band represents the 95% confidence interval of the regression line. Source data are provided as a Source Data file. Source data are provided as a Source Data file.

We next focused on validating spaSim's ability to capture cell co-localization. We compared the values obtained for the real and simulated images of the co-location metrics shown in Table N3.1 (Figure N3.4). In all cases there is a positive correlation between these values, with an R^2 of 0.90 for the APD, 0.33 for the AMD with islet cells as reference cells, 0.08 for the AMD with endothelial cells as reference cells, 0.52 for the CIN, 0.24 for the AUC scores, 0.47 for the MS and 0.49 for the NMS, indicating that the images generated by spaSim generally capture the cell co-localization properties of real images. However, it is best at capturing simpler co-localization metrics, such as the APD, rather than more complex metrics such as the AUC and MS. These likely depend more on cell-cell associations and capturing these is currently beyond the scope of spaSim as assigning the cell types to cells is done randomly.

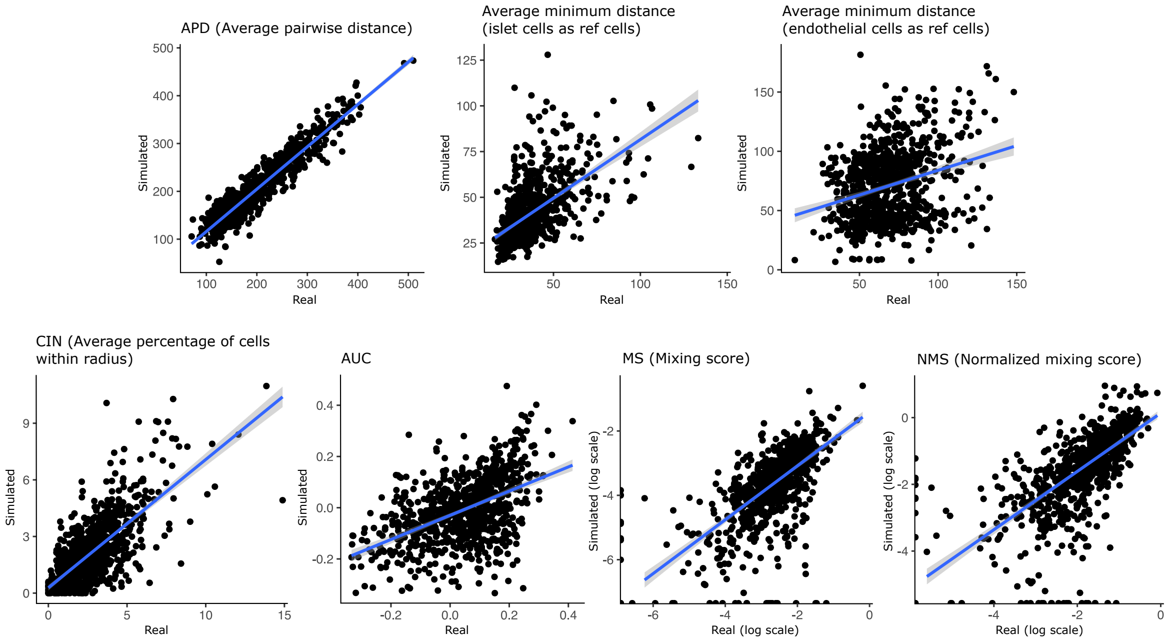

Figure N3.4. Scatterplot of co-localization metrics of the real and simulated images. Metrics for MS and NMS are log scaled. The error band represents the 95% confidence interval of the regression line. Source data are provided as a Source Data file. APD: Average pairwise distance; CIN: Cells In Neighbourhood; AUC: Area Under the Curve of the Cross-K function; MS: Mixing Score; NMS: Normalized Mixing Score

We also compared the ability of spaSim to capture key clustering properties of images, namely the number of cells in islets and the RB-C (Figure N3.5) . Note that spaSim does not directly take in as a parameter the number of clustered cells, but rather the cluster diameter *d*. We therefore estimated *d* from *D* and $N_{islet\text{-}cells}$, which was then used as input for the simulation.

Comparing the number of cells within the islet area in real images and those found in the simulated images, we see a correlation with an R^2 of 0.65, indicating that our process to generate clusters produces clusters of comparable sizes. We also compared the R-BC metrics between the real and simulated images, which resulted in an R^2 of 0.45.

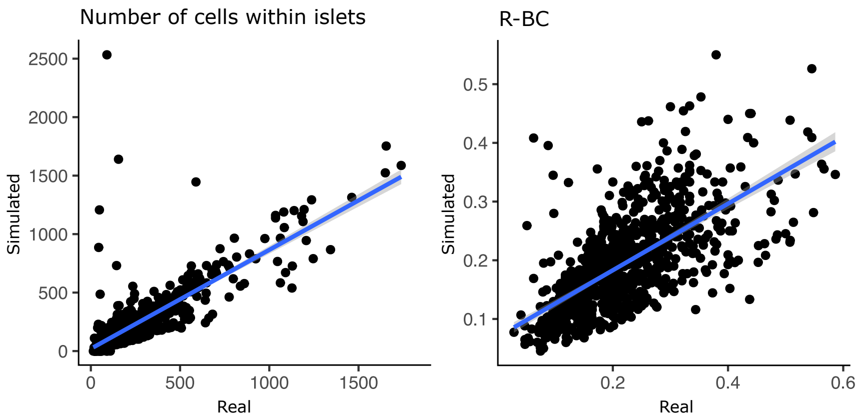

Figure N3.5. Scatterplots of tissue regions-associated metrics of the real and simulated images. The error band represents the 95% confidence interval of the regression line. Source data are provided as a Source Data file. R-BC: Ratio of Bordering cell count to Cluster cell count.

Overall, the simulations generated by spaSim are able to capture co-localization, clustering and the cell type proportions found in real images reasonably well. We note that a perfect correspondence between the spatial metrics of the real and simulated images is not expected, as the simulated images do not include the biological randomness that occurs in real images and capturing cell-cell associations is currently beyond the scope of spaSim. This provides a clean and controlled environment to understand the behavior of spatial metrics.

Note N4

We introduced the Entropy Gradients metric to characterize cell colocalization in Figure 4. A key aspect of the Entropy Gradients is the selection of the reference and target populations, as the results are dependent on their relative number. Here, we discuss the behavior of the Entropy Gradients metric when selecting different cell types as reference populations, and explore other colocalization metrics that can be applied incorporating the concept of "Gradients".

To test the behavior of the Entropy Gradients with different cell types as reference, we simulated a set of five images with different proportions of A and B cells with intuitive 'Attraction' (Figure N4.1A). Images 1, 2, and 3 have A cells as the major population (more A cells than B cells), Image 4 has roughly the same number of A and B cells, and B cells are the major population in Image 5.

First, we performed the Entropy Gradients analysis using B cells as the reference population (Figure N4.1B), as we did in Figure 6. As expected, we observe a drop in the slope near radius = 0 in the first three images (Figure N4.1B). However, in Image 4 the curve becomes flat as there is a similar number of reference and target cells, and finally in Image 5, the slope is reversed as there are more target cells than reference cells. Therefore, defining cells as "Attracting" based on a negative slope as we have proposed is conditional on selecting the minor population as the reference population.

Consistent with this, when using the majority cell type as reference, the trend of the Entropy Gradients is reversed. If we perform the Entropy Gradients analysis using A cells (Figure N4.1C) as the reference population, we obtain a positive slope near radius = 0 in the first three images, followed by a flattening of the curve in Image 4, and then a reversal of the slope in Image 5 (Figure N4.1C).

In cases of repulsion between cells, using the majority population of the reference cells adds an additional level of complexity. For example, we applied the Entropy Gradients using the Cell A as reference to the same set of simulated images as those presented in Figure 4c (Figure N4.2A,B). As expected, in cases of attraction (first two images) when the reference population is the majority cell type, the first slope is negative (first two images). However, in cases of repulsion there is also a negative curve. The key difference here is the change in range of the curve. In the case of attraction, the curve sits consistently around 0.8 and quickly flattens after the initial slope. On the other hand, in cases of repulsion the curve dramatically increases and does not flatten.

Given that using the major population as the reference population would require looking at both the slope and curve range to define attraction and repulsion, we opted to use the minority population as the reference population in our main results as case study, as this provides a more intuitive and tractable understanding of the slopes.

Finally, we also point out that we can use other colocalization metrics, such as NMS instead of entropy with the concept of Gradients (Figure N4.2C,D).

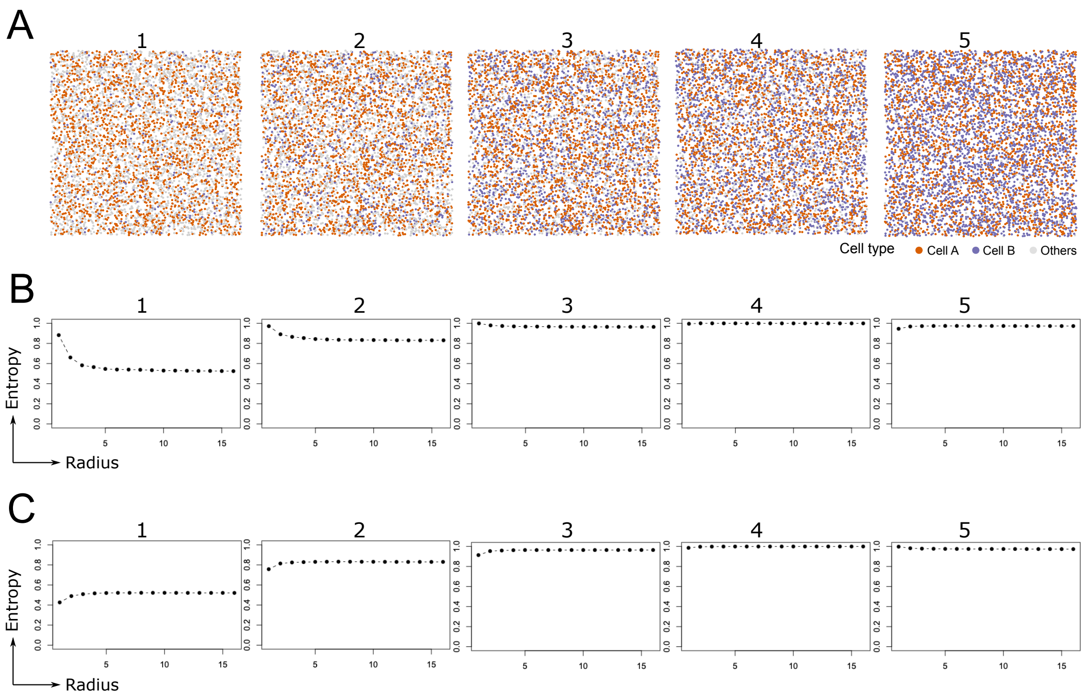

Figure N4.1. Entropy Gradients analysis on simulated A and B cell type mixed images with different cell types as reference. (A) Simulated images by spaSim. **(B)** Entropy Gradient calculation using cells of type B as reference cells. **(C)** Gradient entropy calculation using A cells as reference cells. Source data are provided as a Source Data file.

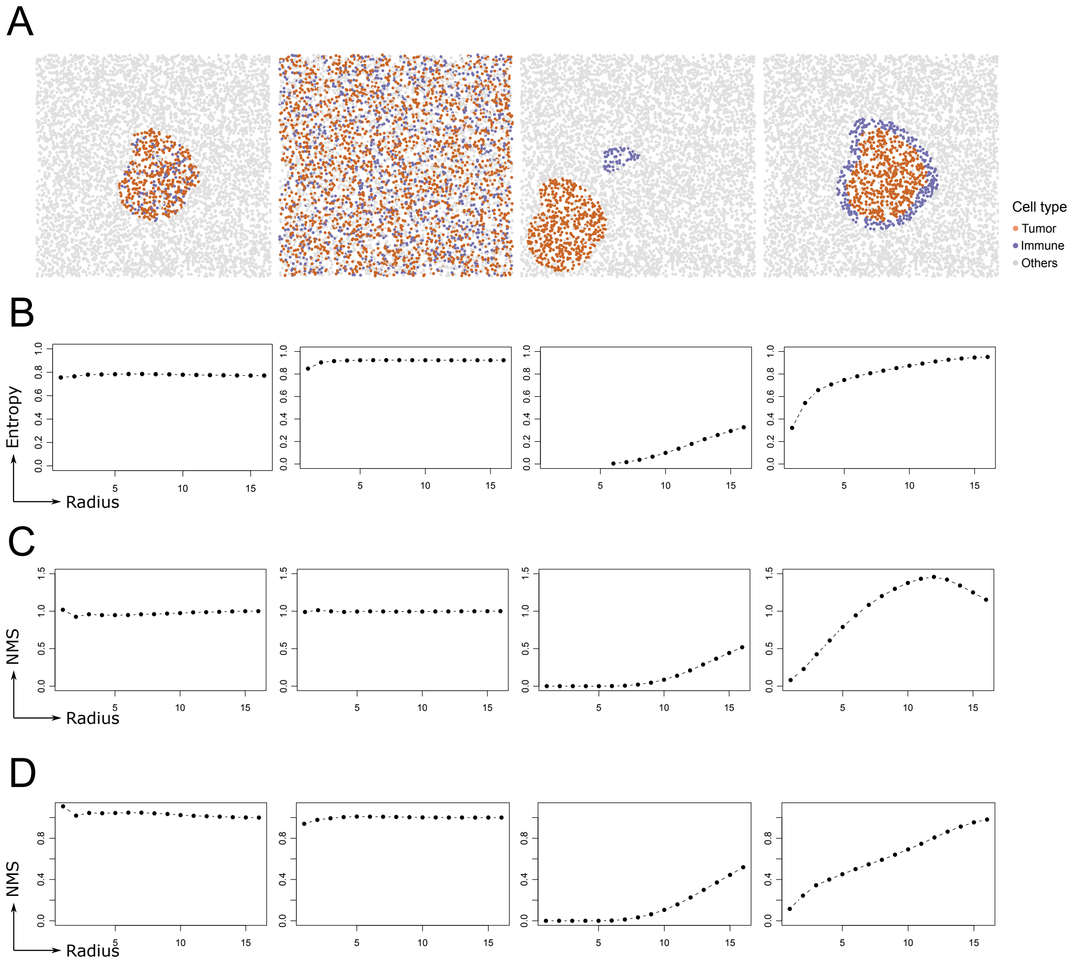

Figure N4.2. Incorporating colocalization metrics with gradients computed over a range of radii. (A) The same simulations shown in Figure 4c. The patterns from left to right are mixing cluster, mixing background, separate clusters and a ring of Tumor cells, respectively. **(B)** Gradient entropy calculation using Tumor cells as reference cells (while the calculation in Figure 4c used Immune cells as reference cells). Each plot corresponds to each image in panel A. **(C)** Using the NMS (normalized mixing score) with gradient computing using Immune cells as reference cells. **(D)** Using the NMS in the calculation of gradients using Tumor cells as reference cells. Similar results as those with the localized entropy are obtained. When using the Tumor cell population as a reference population, a consistent high score across radii is indicative of attraction, whereas a positive slope defined by a large increase in the scores at increasing radii is indicative of repulsion. Source data are provided as a Source Data file.

Note N5

In Figure 6c we identified the tumor bordering cells (which define the tumor margin) and defined regions relative to the tumor areas in three melanoma whole-tissue sections (Figure N5.1). These samples were taken from the same patient at diagnosis, relapse and metastasis. Next, we calculated the proportions of immune populations in each region (Table N5.1), and the summary distances of each tumor population to the identified tumor border (Table N5.2).

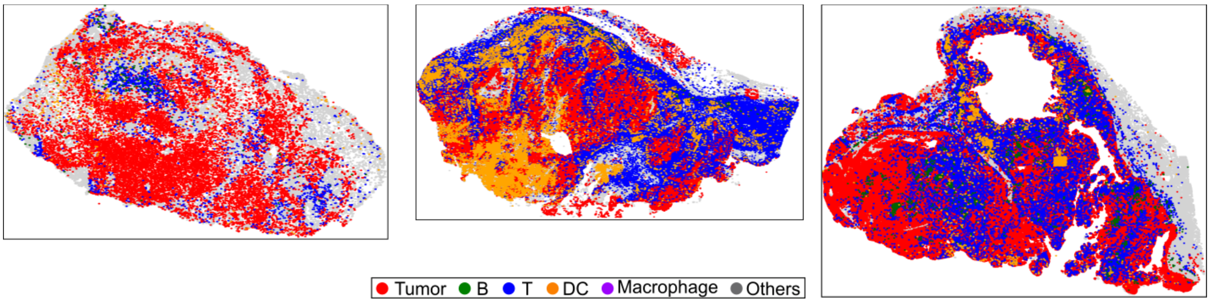

Figure N5.1. Plot cell locations in melanoma images, highlighting the immune populations. The proportions of T cells (blue) are higher than other immune populations in all three images. Immune populations are concentrated inside of the tumor regions in the primary (left) and metastatic melanoma images (right), while in the relapse image (middle), immune populations are concentrated in the stromal area. These observations are consistent with the results calculated by SPIAT (Table N5.1). B: B cells; T: T cells, DC: Dendritic Cells.

Immune populations in the primary and relapse samples included T cells, B cells, dendritic cells (DC), and the metastatic sample included these plus macrophages (Ma). The proportion of each immune population in each tumor region (relative to all cells in the respective region) is low given the high number of tumor cells. Among all immune cell types, T cells are the most common (highlighted in red in Table N5.1). All immune populations are concentrated in the inside of the tumor area in the primary and metastatic samples, and concentrated in the stomal area in the relapse sample (highlighted in blue in Table N5.1). With SPIAT, we can also calculate the distances of infiltrated and stromal immune populations to the tumor margin (Table N5.2).

Table N5.1. Summary of the proportion of immune populations in each defined region in the melanoma samples

Sample name	Cell type	Relative to	Infiltrated	Internal margin	External margin	Stromal
**Primary MEL**	**T**	All cells in the structure	0.0552	0.0657	0.0763	0.0396
**Primary MEL**	**B**	All cells in the structure	0.0048	0.0050	0.0080	0.0039
**Primary MEL**	**DC**	All cells in the structure	0.0080	0.0106	0.0233	0.0225
**Primary MEL**	**T, B, DC**	All cells in the structure	0.0681	0.0804	0.1052	0.0645
**Primary MEL**	**T**	T, B, DC cells in the structure	0.8117	0.8164	0.7252	0.6130
**Primary MEL**	**B**	T, B, DC cells in the structure	0.0699	0.0625	0.0766	0.0609
**Primary MEL**	**DC**	T, B, DC cells in the structure	0.1184	0.1211	0.1982	0.3261
**Primary MEL**	**T**	T in the image	**0.7266**	0.1118	0.0861	0.0754
**Primary MEL**	**B**	B in the image	**0.7134**	0.0976	0.1037	0.0854
**Primary MEL**	**DC**	DC in the image	**0.5690**	0.0891	0.1264	0.2155
**Relapse MEL**	**T**	All cells in the structure	0.0372	0.0731	0.1476	0.2027
**Relapse MEL**	**B**	All cells in the structure	0.0000	0.0001	0.0001	0.0020
**Relapse MEL**	**DC**	All cells in the structure	0.0151	0.0386	0.0942	0.0615
**Relapse MEL**	**T, B, DC**	All cells in the structure	0.0552	0.1118	0.2418	0.2662
**Relapse MEL**	**T**	T, B, DC cells in the structure	0.7106	0.6538	0.6102	0.7616
**Relapse MEL**	**B**	T, B, DC cells in the structure	0.0001	0.0008	0.0003	0.0073
**Relapse MEL**	**DC**	T, B, DC cells in the structure	0.2893	0.3453	0.3894	0.2311
**Relapse MEL**	**T**	T in the image	0.1573	0.0514	0.0628	**0.7285**

**Relapse MEL**	**B**	B in the image	0.0045	0.0090	0.0045	**0.9819**
**Relapse MEL**	**DC**	DC in the image	0.1817	0.0771	0.1138	**0.6274**
**Metastasis MEL**	**T**	All cells in the structure	0.0340	0.0308	0.0527	0.0390
**Metastasis MEL**	**B**	All cells in the structure	0.0029	0.0008	0.0002	0.0014
**Metastasis MEL**	**DC**	All cells in the structure	0.0036	0.0047	0.0050	0.0033
**Metastasis MEL**	**Ma**	All cells in the structure	0.0050	0.0036	0.0041	0.0031
**Metastasis MEL**	**T, B, DC, Ma**	All cells in the structure	0.0441	0.0399	0.0643	0.0490
**Metastasis MEL**	**T**	T, B, DC, Ma cells in the structure	0.7372	0.7709	0.8190	0.8325
**Metastasis MEL**	**B**	T, B, DC, Ma cells in the structure	0.0663	0.0208	0.0382	0.0295
**Metastasis MEL**	**DC**	T, B, DC, Ma cells in the structure	0.0827	0.1179	0.0774	0.0708
**Metastasis MEL**	**Ma**	T, B, DC, Ma cells in the structure	0.1138	0.0904	0.0653	0.0671
**Metastasis MEL**	**T**	T in the image	**0.6692**	0.0433	0.0301	0.2574
**Metastasis MEL**	**B**	B in the image	**0.8372**	0.0162	0.0196	0.1270
**Metastasis MEL**	**DC**	DC in the image	**0.7054**	0.0622	0.0268	0.2057
**Metastasis MEL**	**Ma**	Ma in the image	0.7853	0.0386	0.0183	0.1578

Table N5.2. Summary of the distances of immune population to the tumor margin in the melanoma samples

Sample Name	Cell Type	Location of cell type	Min	Max	Mean	Median	St.dev
**Primary MEL**	**T, B, DC**	Tumor_area	12.35	2178.10	821.80	877.82	543.85
**Primary MEL**	**T, B, DC**	Stroma	13.65	927.61	166.54	102.32	187.99
**Primary MEL**	**T**	Tumor_area	12.35	2178.10	832.37	916.72	552.58
**Primary MEL**	**T**	Stroma	13.65	813.80	150.21	90.73	176.63
**Primary MEL**	**B**	Tumor_area	17.03	2061.18	865.52	950.34	484.28
**Primary MEL**	**B**	Stroma	17.10	253.84	103.03	80.62	70.00
**Primary MEL**	**DC**	Tumor_area	17.24	2085.85	724.10	678.80	506.61
**Primary MEL**	**DC**	Stroma	17.41	927.61	224.51	132.29	221.60
**Relapse MEL**	**T, B, DC**	Tumor_area	10.31	2909.54	483.53	282.22	528.35
**Relapse MEL**	**T, B, DC**	Stroma	11.50	6294.39	1148.27	793.01	1140.39
**Relapse MEL**	**T**	Tumor_area	10.31	2909.54	529.14	305.37	571.90
**Relapse MEL**	**T**	Stroma	12.02	6294.39	1338.07	998.82	1209.74
**Relapse MEL**	**B**	Tumor_area	22.21	1108.61	229.03	54.46	431.85
**Relapse MEL**	**B**	Stroma	24.19	5671.92	1214.18	1036.53	980.95
**Relapse MEL**	**DC**	Tumor_area	10.55	2735.72	379.43	227.34	392.44
**Relapse MEL**	**DC**	Stroma	11.50	6266.71	571.39	382.92	611.42
**Metastasis MEL**	**T, B, DC, Ma**	Tumor_area	11.00	4254.09	1247.79	979.39	983.19
**Metastasis MEL**	**T, B, DC, Ma**	Stroma	11.88	4844.54	490.28	345.31	494.27
**Metastasis MEL**	**T**	Tumor_area	11.00	4254.09	1182.56	912.14	968.22
**Metastasis MEL**	**T**	Stroma	11.88	4451.85	468.51	337.07	445.70
**Metastasis MEL**	**B**	Tumor_area	21.10	4027.38	1697.56	1610.94	973.87

**Metastasis MEL**	**B**	Stroma	23.55	4597.79	491.10	406.00	457.42
**Metastasis MEL**	**DC**	Tumor_area	14.71	4025.84	1338.36	968.88	1027.44
**Metastasis MEL**	**DC**	Stroma	16.98	3519.80	503.29	343.91	586.97
**Metastasis MEL**	**Ma**	Tumor_area	15.70	4054.01	1353.77	1183.41	971.06
**Metastasis MEL**	**Ma**	Stroma	20.50	4844.54	746.23	437.83	806.31

Supplementary References

1. Trigos AS*, et al.* Tumor immune microenvironment of primary prostate cancer with and without germline mutations in homologous recombination repair genes. *Journal for ImmunoTherapy of Cancer* **10**, e003744 (2022).

2. Keren L*, et al.* A Structured Tumor-Immune Microenvironment in Triple Negative Breast Cancer Revealed by Multiplexed Ion Beam Imaging. *Cell* **174**, 1373-1387 e1319 (2018).

3. Pizzolla A*, et al.* Tissue-resident memory T cells from a metastatic vaginal melanoma patient are tumor-responsive T cells and increase after anti-PD-1 treatment. *Journal for ImmunoTherapy of Cancer* **10**, e004574 (2022).

4. Damond N*, et al.* A Map of Human Type 1 Diabetes Progression by Imaging Mass Cytometry. *Cell Metab* **29**, 755-768.e755 (2019).

5. Bortolomeazzi M*, et al.* A SIMPLI (Single-cell Identification from MultiPLexed Images) approach for spatially-resolved tissue phenotyping at single-cell resolution. *Nature Communications* **13**, 781 (2022).

6. Schapiro D*, et al.* histoCAT: analysis of cell phenotypes and interactions in multiplex image cytometry data. *Nature Methods* **14**, 873-876 (2017).

7. Eling N, Damond N, Hoch T, Bodenmiller B. cytomapper: an R/Bioconductor package for visualization of highly multiplexed imaging data. *Bioinformatics* **36**, 5706-5708 (2020).

8. van Maldegem F*, et al.* Characterisation of tumour microenvironment remodelling following oncogene inhibition in preclinical studies with imaging mass cytometry. *Nature Communications* **12**, 5906 (2021).

9. Dries R*, et al.* Giotto: a toolbox for integrative analysis and visualization of spatial expression data. *Genome Biology* **22**, 78 (2021).

10. Somarakis A, Unen VV, Koning F, Lelieveldt B, Höllt T. ImaCytE: Visual Exploration of Cellular Micro-Environments for Imaging Mass Cytometry Data. *IEEE Transactions on Visualization and Computer Graphics* **27**, 98-110 (2021).

11. Opzoomer JW*, et al.* ImmunoCluster provides a computational framework for the nonspecialist to profile high-dimensional cytometry data. *Elife* **10**, (2021).

12. Stoltzfus CR*, et al.* CytoMAP: A Spatial Analysis Toolbox Reveals Features of Myeloid Cell Organization in Lymphoid Tissues. *Cell Rep* **31**, 107523 (2020).

13. Palla G*, et al.* Squidpy: a scalable framework for spatial omics analysis. *Nature Methods* **19**, 171-178 (2022).

14. Mi H*, et al.* Digital Pathology Analysis Quantifies Spatial Heterogeneity of CD3, CD4, CD8, CD20, and FoxP3 Immune Markers in Triple-Negative Breast Cancer. *Frontiers in Physiology* **11**, (2020).
