# Supplementary material for: Spatial analysis with SPIAT and spaSim to characterize and simulate tissue microenvironments
Source: Nat Commun. 2023 May 15;14:2697. doi: 10.1038/s41467-023-37822-0 (PMC10185501; doi:10.1038/s41467-023-37822-0)
Supplement: Supplementary file 3 — Reporting Summary [file 41467_2023_37822_MOESM3_ESM.pdf]

nature portfolio

Corresponding author(s): Anna Trigos

Last updated by author(s): 14/03/2022

Reporting Summary

Nature Portfolio wishes to improve the reproducibility of the work that we publish. This form provides structure for consistency and transparency in reporting. For further information on Nature Portfolio policies, see our Editorial Policies and the Editorial Policy Checklist.

Statistics

For all statistical analyses, confirm that the following items are present in the figure legend, table legend, main text, or Methods section.

n/a	Confirmed	
☐	☒	The exact sample size (*n*) for each experimental group/condition, given as a discrete number and unit of measurement
☐	☒	A statement on whether measurements were taken from distinct samples or whether the same sample was measured repeatedly
☐	☒	The statistical test(s) used AND whether they are one- or two-sided<br>*Only common tests should be described solely by name; describe more complex techniques in the Methods section.*
☒	☐	A description of all covariates tested
☐	☒	A description of any assumptions or corrections, such as tests of normality and adjustment for multiple comparisons
☐	☒	A full description of the statistical parameters including central tendency (e.g. means) or other basic estimates (e.g. regression coefficient) AND variation (e.g. standard deviation) or associated estimates of uncertainty (e.g. confidence intervals)
☐	☒	For null hypothesis testing, the test statistic (e.g. *F*, *t*, *r*) with confidence intervals, effect sizes, degrees of freedom and *P* value noted<br>*Give P values as exact values whenever suitable.*
☒	☐	For Bayesian analysis, information on the choice of priors and Markov chain Monte Carlo settings
☒	☐	For hierarchical and complex designs, identification of the appropriate level for tests and full reporting of outcomes
☒	☐	Estimates of effect sizes (e.g. Cohen's *d*, Pearson's *r*), indicating how they were calculated

Our web collection on statistics for biologists contains articles on many of the points above.

Software and code

Policy information about availability of computer code

Data collection	No software was used for data collection
Data analysis	The SPIAT package is available at: https://github.com/TrigosTeam/SPIAT as well as in Bioconductor (https://bioconductor.org/packages/SPIAT/). The SPIAT tutorial is available at: https://trigosteam.github.io/SPIAT/. The spaSim package is available at: https://github.com/TrigosTeam/spaSim as well as in Bioconductor (https://bioconductor.org/packages/spaSim/). The spaSim tutorial is available at: https://trigosteam.github.io/spaSim/. The data analysis and simulation code to reproduce all results of the manuscript is available at: https://github.com/TrigosTeam/SPIATspaSimNCCodeShare.<br><br>For the prostate cancer dataset, the inForm Advanced Image Analysis Software (PerkinElmer, versions 2.3 and 2.4) was used to process raw microscopy images and extract cell coordinates and marker intensities.<br>For the melanoma dataset, we used the inForm version 2.4.8 and the HALO image analysis platform (Indica Labs version 3.0.311) with the HighPlex v2.0 module to process raw microscopy images and extract cell coordinates and marker intensities.<br>For spatstat analyses, we used spatstat.random v3.0-1.

For manuscripts utilizing custom algorithms or software that are central to the research but not yet described in published literature, software must be made available to editors and reviewers. We strongly encourage code deposition in a community repository (e.g. GitHub). See the Nature Portfolio guidelines for submitting code & software for further information.

Data

Policy information about availability of data

All manuscripts must include a data availability statement. This statement should provide the following information, where applicable:

- Accession codes, unique identifiers, or web links for publicly available datasets
- A description of any restrictions on data availability
- For clinical datasets or third party data, please ensure that the statement adheres to our policy

The MIBI TNBC data used in this study are available at https://www.angelolab.com/mibi-data. The CODEX colon cancer data used in this study are available at https://data.mendeley.com/datasets/mpjzbtfgfr/1. The IMC diabetes data used in this study are available at https://data.mendeley.com/datasets/cydmwsfztj/1 and https://data.mendeley.com/datasets/cydmwsfztj/2. The prostate cancer and melanoma data are available under restricted access as our patient consent does not allow depositing data online, access can be obtained by contacting the corresponding author.

Field-specific reporting

Please select the one below that is the best fit for your research. If you are not sure, read the appropriate sections before making your selection.

☒ Life sciences ☐ Behavioural & social sciences ☐ Ecological, evolutionary & environmental sciences

For a reference copy of the document with all sections, see nature.com/documents/nr-reporting-summary-flat.pdf

Life sciences study design

All studies must disclose on these points even when the disclosure is negative.

Sample size	No sample size calculation were performed in this study. The number of datasets used in the study was selected to show the diversity of capabilities of the tool across different tissue contexts and diseases. All samples of the datasets were included (except where mentioned in "Data exclusions") to capture as much heterogeneity as possible.
Data exclusions	We only included in our border detection algorithm example prostate cancer images with at least 300 immune and tumour cells. This ensured a large enough number of cells to allow meaningful spatial patterns. For analysis of the distances of endothelial cells to islets, we only used images with at least 10 endothelial cells. Although the original paper of the breast cancer dataset states 41 patients (Keren et al. 2018), there was only data available online for 40. In Figure 4, samples without the relevant cell types were excluded. Colon cancer samples from the CODEX dataset without B cells were excluded from the calculation of the ANNI for B cells.
Replication	All findings can be reproduced. The data analysis and simulation code to reproduce all results of the manuscript is available at: https://github.com/TrigosTeam/SPIATspaSimNCCodeShare.
Randomization	Randomization was not applicable to the study as we did not perform sample collection. Samples from the datasets had been previously classified by the original authors based on the known biology of each sample or the clinical characteristics of the patients.
Blinding	Blinding was not applicable because all samples of each of the datasets were analysed with the same automated pipeline.

Reporting for specific materials, systems and methods

We require information from authors about some types of materials, experimental systems and methods used in many studies. Here, indicate whether each material, system or method listed is relevant to your study. If you are not sure if a list item applies to your research, read the appropriate section before selecting a response.

Materials & experimental systems

n/a	Involved in the study	
☒	☐	Antibodies
☒	☐	Eukaryotic cell lines
☒	☐	Palaeontology and archaeology
☒	☐	Animals and other organisms
☐	☒	Human research participants
☒	☐	Clinical data
☒	☐	Dual use research of concern

Methods

n/a	Involved in the study	
☒	☐	ChIP-seq
☒	☐	Flow cytometry
☒	☐	MRI-based neuroimaging

Human research participants

Policy information about studies involving human research participants

Population characteristics	From Trigos et al 2022: Prostate cancer tissue from the prostate was obtained from 26 male patients diagnosed with prostate cancer. Patients were germline tested for alterations in DNA repair genes, and were verified to not have any of these alterations.<br>From Pizzolla et al 2022: Melanoma tissue was obtained from a female patient with stage 3 metastatic vaginal melanoma. Serial samples were collected from the primary site, as well as a metastatic and relapse site.<br>From Keren et al 2018: Breast cancer tissue was obtained from female patients with triple-negative breast cancer. These patients did not belong to any specific subtype (ER and PR positivity less than 1%, HER2 unamplified and Her2 not 3)<br>From Schurch et al 2020: 35 colorectal cancer cases (17 with Crohn-like reaction pathology [10 females, 7 males] versus 18 Diffuse Inflammatory Infiltration [8 females, 10 males]) were selected at random, matched for gender, age, and cancer type, location, and cancer stage<br>From Damond et al 2019: 12 patients were selected to represent recent onset type-1 diabetes (<0.5 years) (4 patients), long-standing type-1 diabetes (≥8 years) (4 patients), and non-diabetes control (4 patients). Patients were matched by age and gender. Each group was composed of three male and one female patient.<br>No sex or gender analysis was carried out as this was outside the scope of the current work.
Recruitment	No recruitment of participants was performed for this study as all data used was generated in the context of other published work. Namely, Trigos AS, et al. Journal for ImmunoTherapy of Cancer 10, e003744 (2022); Pizzolla A, et al. Journal for ImmunoTherapy of Cancer 10, e004574 (2022); Keren L, et al. A Cell 174, 1373-1387 e1319 (2018); Schürch CM, et al. Cell 182, 1341-1359.e1319 (2020); Damond N, et al. Cell Metab 29, 755-768.e755 (2019).
Ethics oversight	The current work used previously published data, which had the Ethics oversights of the corresponding institutes as outlined in the previously published manuscripts. Namely, Trigos AS, et al. Journal for ImmunoTherapy of Cancer 10, e003744 (2022); Pizzolla A, et al. Journal for ImmunoTherapy of Cancer 10, e004574 (2022); Keren L, et al. A Cell 174, 1373-1387 e1319 (2018); Schürch CM, et al. Cell 182, 1341-1359.e1319 (2020); Damond N, et al. Cell Metab 29, 755-768.e755 (2019).

Note that full information on the approval of the study protocol must also be provided in the manuscript.
